# The use of functional near-infrared spectroscopy in tracking neurodevelopmental trajectories in infants and children with or without developmental disorders: a systematic review

**DOI:** 10.3389/fpsyt.2023.1210000

**Published:** 2023-09-14

**Authors:** Wan-Chun Su, Rebekah Colacot, Nora Ahmed, Thien Nguyen, Tony George, Amir Gandjbakhche

**Affiliations:** Eunice Kennedy Shriver National Institute of Child Health and Human Development (NICHD), National Institutes of Health, Bethesda, MD, United States

**Keywords:** functional near-infrared spectroscopy, neurodevelopment, trajectory, longitudinal study, developmental disorders

## Abstract

Understanding the neurodevelopmental trajectories of infants and children is essential for the early identification of neurodevelopmental disorders, elucidating the neural mechanisms underlying the disorders, and predicting developmental outcomes. Functional Near-Infrared Spectroscopy (fNIRS) is an infant-friendly neuroimaging tool that enables the monitoring of cerebral hemodynamic responses from the neonatal period. Due to its advantages, fNIRS is a promising tool for studying neurodevelopmental trajectories. Although many researchers have used fNIRS to study neural development in infants/children and have reported important findings, there is a lack of synthesized evidence for using fNIRS to track neurodevelopmental trajectories in infants and children. The current systematic review summarized 84 original fNIRS studies and showed a general trend of age-related increase in network integration and segregation, interhemispheric connectivity, leftward asymmetry, and differences in phase oscillation during resting-state. Moreover, typically developing infants and children showed a developmental trend of more localized and differentiated activation when processing visual, auditory, and tactile information, suggesting more mature and specialized sensory networks. Later in life, children switched from recruiting bilateral auditory to a left-lateralized language circuit when processing social auditory and language information and showed increased prefrontal activation during executive functioning tasks. The developmental trajectories are different in children with developmental disorders, with infants at risk for autism spectrum disorder showing initial overconnectivity followed by underconnectivity during resting-state; and children with attention-deficit/hyperactivity disorders showing lower prefrontal cortex activation during executive functioning tasks compared to their typically developing peers throughout childhood. The current systematic review supports the use of fNIRS in tracking the neurodevelopmental trajectories in children. More longitudinal studies are needed to validate the neurodevelopmental trajectories and explore the use of these neurobiomarkers for the early identification of developmental disorders and in tracking the effects of interventions.

## 1. Introduction

Neurodevelopmental trajectories, referring to the sequential patterns of changes in neural systems associated with developmental changes in behaviors over time, have significant clinical and research implications (1). With the advancement of neuroimaging tools, an increasing number of researchers have utilized longitudinal (i.e., measured neural activity at multiple time points) or cross-sectional designs (i.e., measured in different age groups) to study neurodevelopmental trajectories in children with or without developmental disorders (2–4). Using more standard neuroimaging tools such as functional magnetic resonance imaging (fMRI) and electroencephalogram (EEG), studies provided a deeper understanding of the neural mechanisms underlying skill development and suggested typical developmental trajectories (3, 5). For instance, a comprehensive systematic review that gathered 22 years of fMRI findings suggested an age-related increase in Blood Oxygen Level Dependent (BOLD) responses over the semantic processing and lower-level motor and sensory regions, coupled with an age-related decrease over higher-level control regions, indicating increased automaticity of language processing (3). A systematic review and meta-analysis of EEG studies that focused on the development of executive functioning also reported a decreased N2 (i.e., a negative going event-related potential at fronto-central sites, occurs between 200 and 300 mini-seconds after the stimuli presentation) amplitude and latency from 2 to 12 years of age, and the results were associated with their inhibitory control performance (5).

In addition to delineating typical developmental trajectories, it is equally vital to understand atypical neurodevelopmental trajectories in children diagnosed with neurodevelopmental disorders. These disorders encompass various conditions characterized by aberrant development in brain functions, leading to challenges in sensory, motor, and/or cognitive difficulties, etc. (6). For examples, within this classification, children with Autism Spectrum Disorder (ASD) experience difficulties in social communication and the demonstrate of repetitive behaviors (DSM-V; 7); children diagnosed with Attention-Deficit/Hyperactivity Disorder (ADHD) demonstrate persistent pattern of inattention, hyperactivity, and/or of impulsivity (DSM-V; 7); while children with dyslexia struggle to reconcile spelling patterns and with word pronunciations (DSM-V; 7). Neuroimaging studies hold great potential in predicting developmental outcomes and identifying early markers for a range of neurodevelopmental disorders (2, 8, 9). A comprehensive systematic review suggested that infants with an elevated risk of developing ASD showed distinct developmental trajectories in global connectivity (a correlation-based approach to measuring the synchronization in neural activity across widely distributed brain regions) using fMRI, atypical lateralization when processing facial and auditory information using EEG, and varied functional connectivity and regional hemoglobin responses using functional Near-Infrared Spectroscopy (fNIRS) (8). Furthermore, these neural imaging signatures are correlated with ASD symptoms and developmental performance at a later age (8). In short, neuroimaging studies investigating typical and atypical developmental trajectories provide valuable insights into the neural mechanisms underlying developing skills and have important clinical implications such as early identification of neurodevelopmental disorders and outcome prediction.

Systematic reviews of fMRI and EEG studies have provided valuable insights into the neurodevelopmental trajectories of important developmental domains, such as language (3, 10), social (4), and executive functioning (7). However, both fMRI and EEG have their limitations. Due to the constraint of the fMRI scanners and the intolerance of movement artifacts, there are challenges in using fMRI in infants and children with developmental disorders (11). EEG is more child-friendly, however, it provides no structural information and may have poor adaptation to the rapid head growth in infants (12). fNIRS is a non-invasive neuroimaging tool that measures the hemodynamic responses of cortical regions (13). The system consists of emitter and receiver pairs, wherein emitters project infrared light through the skull, forming a banana-shaped arc that extends to the cortical areas. The attenuation of infrared light was used to calculate the changes in concentrations of oxygenated (HbO) and deoxygenated hemoglobin (HbR) chromophores using the Modified Beer–Lambert Law (13). This methodology facilitates the estimation of fNIRS activation, manifested by an increase in HbO concentration and a decrease in HbR concentration. Despite its limitation, including limited penetration depth, lower temporal resolution, and a lack of structural information, fNIRS offers the advantage of only requiring a cap, having better tolerance of movement artifacts, and providing better spatial resolution compared to EEG (13). Moreover, fNIRS allows naturalistic interactions starting from very early in life, making it a promising tool for studying neurodevelopmental trajectories (13). For example, using fNIRS, Lloyd-Fox et al. investigated neurodevelopmental trajectories in typically developing infants starting from neonate to 24 months old (14). Additionally, fNIRS has been used to measure hemodynamic changes (HbO and HbR concentrations) that reflect brain activity during naturalistic social interactions, such as interpersonal synchrony (15, 16) and collaborative/ competitive tasks (17). Given these advantages, fNIRS is an ideal neuroimaging tool to study neurodevelopment. It holds the potential to provide new insights about neural mechanisms, suggest neurobiomarkers for screening infants and children for developmental disorders, and serve as an objective measure for the impacts of interventions. However, to our knowledge, there has been no systematic review summarizing the typical and atypical neurodevelopmental trajectories in infants and children using this relatively novel neuroimaging tool. Hence, the current systematic review summarized the findings from fNIRS studies and suggests potential research directions and clinical implications based on the findings.

## 2. Materials and methods

We conducted literature searches from four allied databases, including PubMed, PsycINFO, Scopus, and Web of Science. We included search terms in two areas, (a) Functional Near Infrared Spectroscopy-related such as “near-infrared spectroscopy” and “fNIRS,” as well as (b) Time-related, such as “longitudinal,” “trajectory,” “age groups,” ‘age-related’, etc. Please see detailed search terms in Supplementary Table S1. The literature was included upon fulfilling the inclusion criteria: (a) Used fNIRS to measure hemodynamic changes in human brains; (b) Included multiple age groups (cross-sectional) or conducted fNIRS visits at multiple time points (Longitudinal) and presented age-related findings in the result section; and (c) included more than one participant. The literature was excluded based on the exclusion criteria: (a) Only included adults or elderly populations (>18 years old); (b) Were review papers, case reports, and protocol papers; (c) Written in languages other than English; (d) Were gray literature including theses and dissertations; (e) Only studied the progression of a disorder or the effects of an intervention.

**Figure 1:**
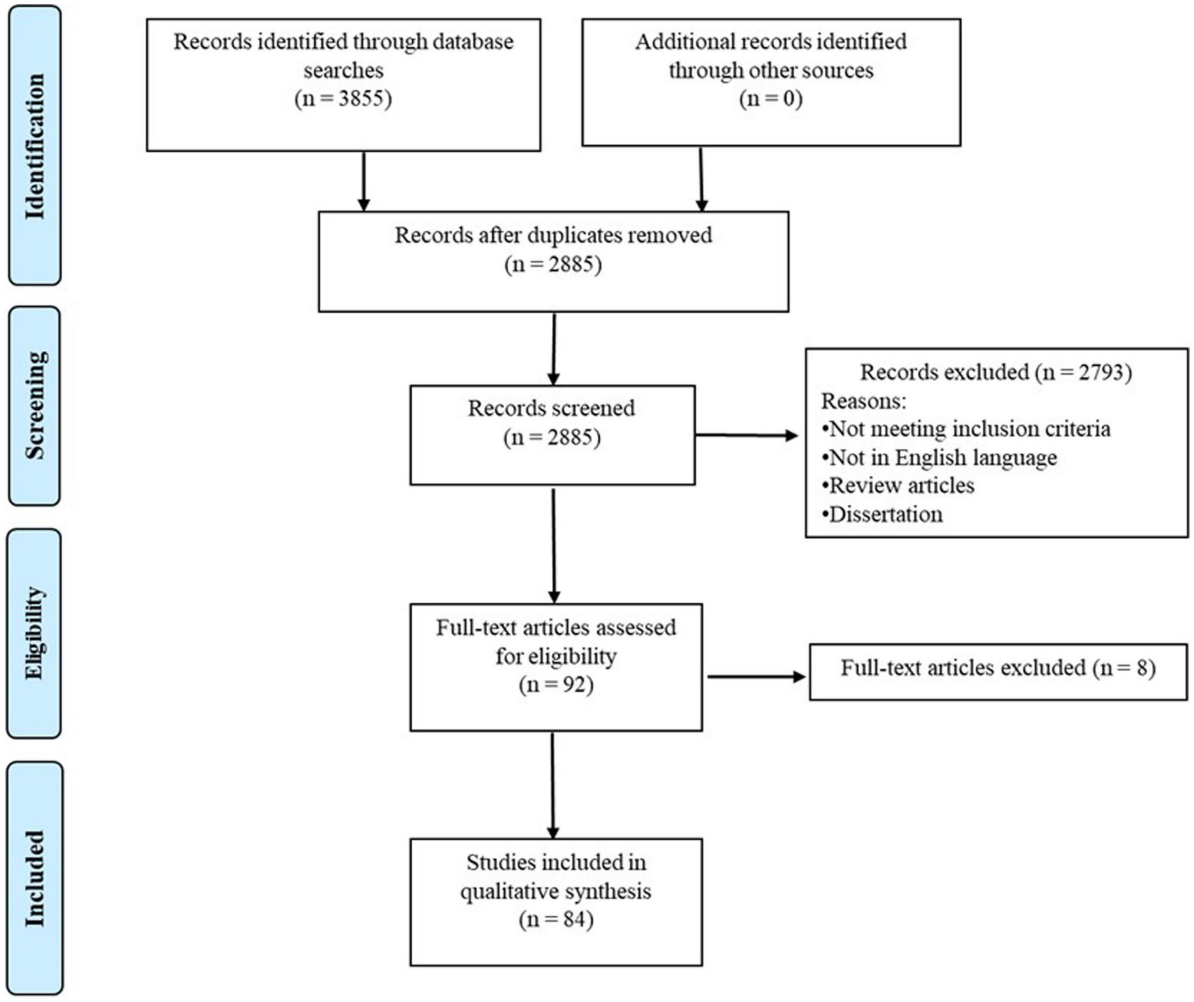
PRISMA diagram for search process.

The final search of the present review was conducted on Feb 6th, 2023, with a result of 3,855 articles in total (1,389 from PubMed, 843 from ProQuest, 1,300 from Scopus, and 323 from Web of Science). After removing duplications, 2,885 articles remained. Two coders (RC and NA) independently screened the literature with 95.3% agreement on the literature eligibility. Disagreements between the two coders were resolved through the consensus meeting with the third coder (WS). Based on the inclusion and exclusion criteria, a total of 84 papers were included in the current review. Please see the PRISMA flow diagram for the search process in Figure 1. In pursuit of our systematic review’s goal to consolidate fNIRS-related neurodevelopmental findings and propose clinical implications, we extracted notable findings, as well as crucial information, including study design, subject characteristics, and fNIRS-related experimental paradigm from the original studies. For example, we extracted comprehensive subject profiles for clinical relevance, encompassing age ranges at each visit, birth history, diagnoses, assessment tools to confirm the diagnosis, sibling status, as well as comorbidity. To enhance the transparency and reproducibility for future researchers, we systematically distilled study design (i.e., longitudinal and cross-sectional approaches, times and number of visits), and fNIRS experimental paradigm (e.g., fNIRS tasks, measures, data processing and analysis methods). This comprehensive extraction process serves to provide a thorough understanding of the reviewed studies, thereby providing a robust foundation for clinicians and researchers to interpret the findings. Additionally, we used the Quality Assessment Tool for Observational Cohort and Cross-Sectional Study from the National Institute of Health to assess the quality and risk of bias of the included paper (Link: https://www.nhlbi.nih.gov/health-topics/study-quality-assessment-tools). According to Li et al., the included studies will be classified into “strong” (≥80%), “good” (70–79%), “fair” (60–69%), or “poor” (<60%) methodological quality based on their quality assessment score (18). Please see the assessment criteria in Supplementary Table S2. Two coders (NA and TG) independently screened the literature with 91% agreement on the quality score. Disagreements between the two coders were resolved through the consensus meeting with the third author (WS).

## 3. Results

### 3.1. Quality assessment

Based on the quality assessment (Supplementary Table S2), 2 papers were rated as “strong,” 7 were rated as “Good,” 34 were rated as “Fair,” and 41 were rated as “Poor” (Supplementary Table S3). The collective evaluation yielded an average score of 62.6, with a standard deviation of 7.3. For a detailed breakdown of the quality assessment scores assigned to each included paper, kindly refer to Supplementary Table S3.

### 3.2. Study and sample characteristics

**Figure 2:**
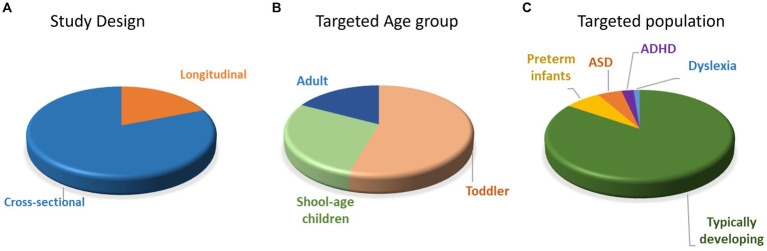
Distribution of papers with different study designs **(A)**, targeted age groups **(B)**, and populations **(C)**. ASD = autism spectrum disorder; ADHD = attention-deficit/hyperactivity disorder.

Of the 84 included articles, 15 studies used longitudinal, 67 studies used cross-sectional, and 2 used both longitudinal and cross-sectional study designs (Figure 2A). The findings from longitudinal studies were similar to that of the cross-sectional studies (Supplementary Tables S4–S9). Nevertheless, it is crucial to recognize that while longitudinal studies propose causal relationships in neurodevelopment, cross-sectional studies primarily indicate developmental patterns devoid of causal inferences. For the longitudinal studies, the number of visits ranged from 2 to 5 (mean ± SD = 2.87 ± 1.13) and the time gaps between visits ranged from 1 to 24 months (mean ± SD = 6.01 m ± 5.81 m). For the cross-sectional study, the number of age groups ranged from 1 to 6 (mean ± SD = 2.44 ± 1.08). The targeted age ranged from 1.5 to 222.6 months (mean ± SD = 72.58 m ± 77.2 m), and the sample sizes ranged from 6 to 254 participants (mean ± SD = 64.89 ± 46.57). Out of 84 studies, 60 studies focused on infant and toddler populations (0–5 years old), 31 studies focused on the school age children and adolescents (6–18 years old), and 19 studies compared the neural development between child and adult populations (22 studies included multiple age group –18 with two groups and 4 with three, Figure 2B). Most studies only included Typically Developing (TD) infants/children/adults (n = 71), 6 studies included preterm infants, 4 studies included infants at risk for (n = 2) or children with ASD (n = 2), 2 studies included children with ADHD, and 1 study included children with Dyslexia (Figure 2C). Specifically, infants were classified as high-risk ASD if they had siblings with ASD diagnoses (19, 20). Among the 2 studies that centered on children with ASD, diagnostic confirmation involved medical records and assessment tools such as Autism Diagnostic Observation Schedule (ADOS) and Childhood Autism Rating Scale (CARS), without specific reference to comorbidities (21, 22). In the case of the 2 studies focusing on children with ADHD, diagnoses were confirmed by pediatric neurologists based on DSM-IV or DSM-V (23, 24). One study ruled out comorbidities such as ASD and learning disabilities (23), while the other included children with dyslexia while excluding ASD comorbidity (24). Lastly, the sole study focusing on children with dyslexia did not specify their diagnostic confirmation method (25).

### 3.3. fNIRS experimental setup

Most of the included studies used a commercial fNIRS system from companies including: Hitachi (Hitachi ETG-100: 8.64%, Hitachi ETG-4000: 27.16%, Hitachi ETG 7000: 11.11%), NIRX (NIRScout: 6.27%, DYNOT: 2.47%, NIRsport: 1.23%), TechEn (CW4: 1.23%, CW6: 8.64%), Shimadzu (FOIRE: 2.47%), ISS (Imagent: 3.70%), Spectratech (OEG-16: 6.17%, OEG-SpO2: 1.23%), Hamamatsu Photonics (NIRO-200: 4.94%, NIRO-300: 1.23%), Gowerlabs (NTS: 3.70%), University College London (UCL-fNIRS: 3.70%), Edmund Optics (NIRSmart: 1.23%), and fNIR Devices (fNIR device 201C: 2.47%). Two studies used OMM-1090S (26, 27), one used Omniat Tissue Oxymeter (28), and one used a custom-built fNIRS device (i.e., fNIRS-CBCD) (14). The number of channels ranged from 1 to 94 (mean ± SD = 27.20 ± 20.21) and covered the regions including the frontal, temporal, parietal, and occipital lobes.

The processing procedures varied across the studies, albeit with a common thread. A prevailing strategy encompassed conducting data analysis on raw light intensity in the majority of cases. Noise data was manually eliminated based on video and data visualization, followed by the implementation of preprocessing methods like filtering (e.g., employing bandpass filters) and the mitigation of motion artifacts (e.g., through Principal Component Analysis). The resultant preprocessed light intensities were subsequently translated into concentrations of HbO and HbR via the adapted Beer–Lambert Law. In studies concentrating on connectivity, the prevailing practices involved employing correlations and phase analyses as initial steps prior to the application of rigorous statistical analyses. Conversely, in activation-focused investigations, the General Linear Model emerged as a commonly utilized tool for estimating the Hemodynamic Response Function (HRF), often in conjunction with blocking or baseline correction techniques. Please refer to Supplementary Figure S1 for the common data processing pipeline. It’s noteworthy that a substantial proportion of studies (66%) opted for the utilization of HomER 2^1^ as their data processing software. The array of statistical analysis methods comprised prominent techniques such as ANOVA (68%), Pearson/Spearman correlation (49%), and Regressions (29%).

**Figure 3:**
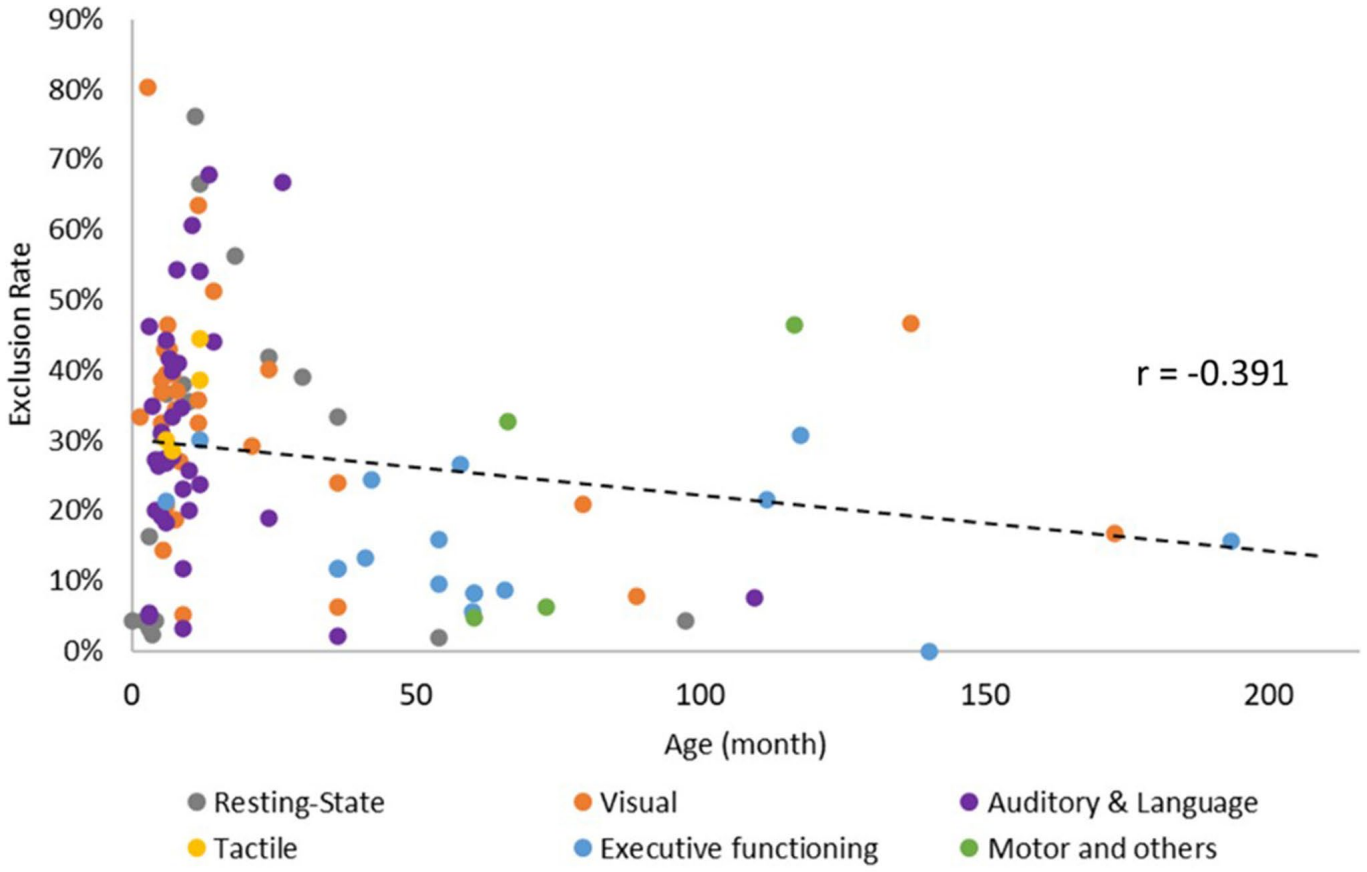
Correlation between age and exclusion rate of fNIRS data.

Sixteen studies focused on the neural activity during resting-state, and the time of recording ranged from 8 to 900 s (mean ± SD = 452.53 s ± 247.25 s). The rest of the studies included multiple tasks/conditions; 28.57% arranged the conditions using set-order, 3.57% arranged them alternatively, 19.64% counterbalanced the occurrence of the condition, 21.43% were pseudo-randomized, and 26.79% randomized the order of the task/condition. The number of trials ranged from 1 to 64 (mean ± SD = 14.17 ± 14.58). Within each trial, the time for baseline ranged from 1 to 42.5 s (mean ± SD = 15.11 s ± 9.88 s), and the time of stimulation ranged from 1 to 450 s (mean ± SD = 21.85 s ± 55.20s). Lastly, the exclusion rate ranged from 0 to 54.36% (mean ± SD = 25.96% ± 16.74%). Using Pearson correlation, we found significant negative correlation between age and exclusion rate (r = −0.391, *p* < 0.05), with studies including younger infants showing higher and more variable exclusion rates (Figure 3).

### 3.4. Domains- specific neurodevelopmental trajectories

**Figure 4:**
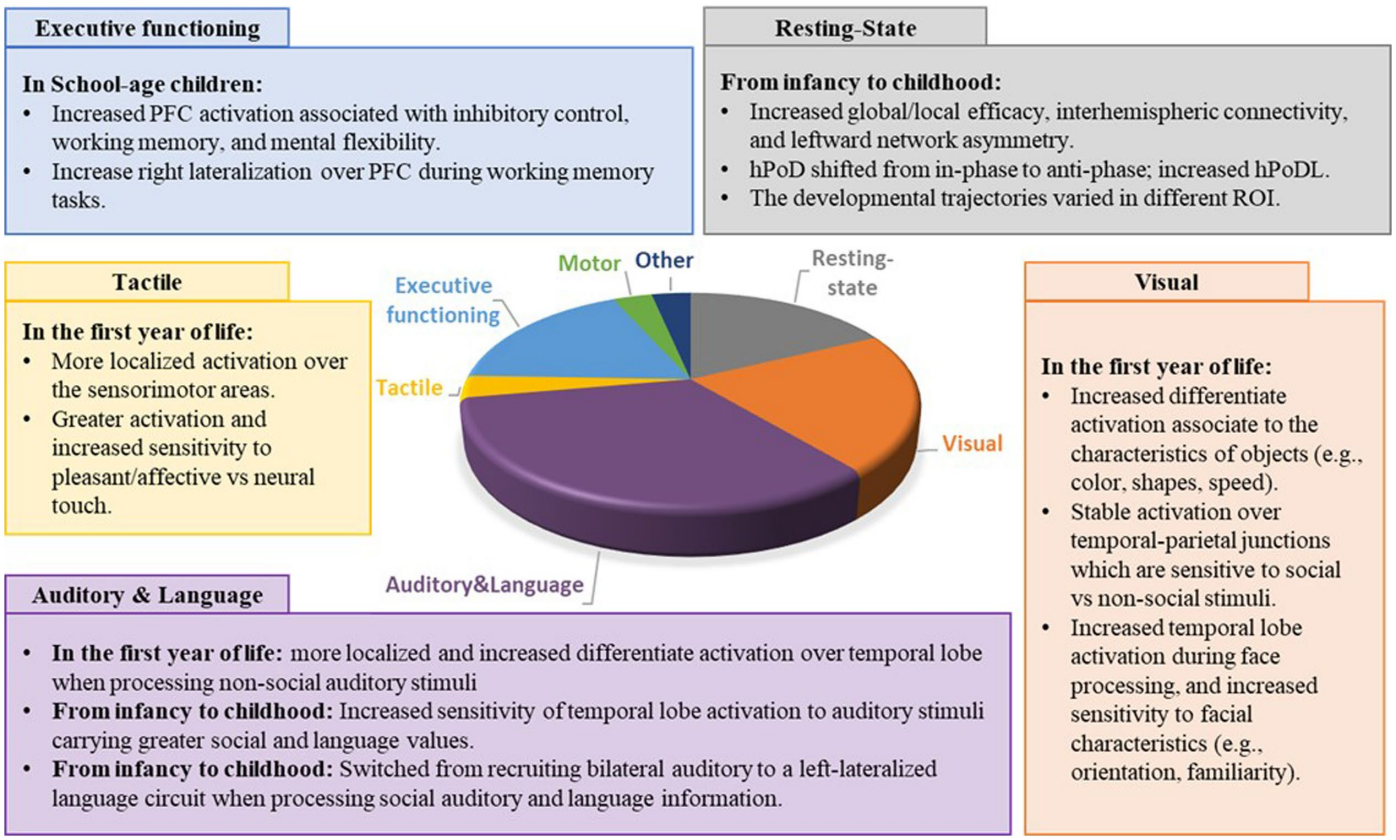
Distribution and main findings of the papers focused on different developmental domains. PFC = prefrontal cortex; ROI = region of interest; HPod, Hemoglobin phase; HPoDL, Phase locking index of HPoD.

Out of 84 articles, 16 focused on the neurodevelopmental trajectories during resting-state, 19 focused on visual related information processing, 30 focused on auditory information processing and language, 3 focused on the tactile information processing, 16 focused on executive functioning, 3 focused on motor-related tasks, and 3 focused on other tasks (i.e., arithmetic, gratification, creativity). Six articles focused on more than one domain. Please see Figure 4 for the distribution of papers focusing on different domains.

#### 3.4.1. Resting-state

Sixteen studies investigated the functional connectivity and the haemoglobin phase of HbO and HbR during resting-state (19–21, 25, 29–40). Eight studies focused on TD children, 4 compared developmental trajectories between full-term and pre-term infants, and 4 examined children with or at risk for developing ASD and dyslexia (Supplementary Table S4). In typical development, age-related increases were observed in global/local efficiency (i.e., the capacity of information transferring within a brain network) (29, 30), interhemispheric connectivity (i.e., the connectivity between two hemispheres) (31, 32), and leftward network asymmetry (i.e., the differences in the ability of information segregation and integration between the two hemispheres) (29, 33). Moreover, the average phase difference between the instantaneous phases of HbO and HbR(hPod) shifted from in-phase to anti-phase, the degree of phase locking (hPod_L_) between HbO and HbR, and the complexity of hemodynamic oscillation increased from infancy to adulthood and decreased in elderly age (34–37). Specifically, Liu et al. found similar local efficiency but greater global efficiency over the left hemisphere in infants aged 6 ~ 9 months compared to those aged 3 ~ 6 months (29). Later in life, Cai et al. found increased local efficiency from adolescence to adulthood (11 ~ 27 years) and increased global efficiency from childhood to adolescence (7 ~ 13 years) (30). For interhemispheric connectivity, Homae et al. (31) found increased interhemispheric connectivity over the temporal, parietal, and occipital regions from neonate to 6 months old infants (31); while Bulgarelli et al. found increased frontal temporoparietal interhemispheric connectivity between 11 to 18 months, with connectivity remaining stable from 18 to 36 months (32). Regarding network asymmetry, Liu et al. found increased leftward asymmetry over the frontal and temporal regions in infancy (3 to 9 months) (29); while Cai et al. found increased leftward asymmetry over the frontal, parietal, and occipital regions from childhood to adulthood (7 to 27 years) (33). Lastly, for phase oscillation, Taga et al. found a shift of hPoD values over frontal, left temporal, and occipital regions from in-phase to anti-phase and an increased hPoD_L_ value over the frontal and left temporal regions in the first year of life (neonate to 11 months) (34, 35). Tracking the phase differences between hemodynamic oscillations throughout the life span, Liang et al. found increased hPod, hPod_L_, and complexity of hemodynamic oscillations from infancy to adulthood (neonate to 27 years), which decreased during elderly ages (58 to 77 years) (36, 37).

Despite overall consistent developmental trajectories in TD children, the developmental changes in different cortical regions vary (Supplementary Table S4) (30–32, 38). For example, Homae et al. found age-related increases in resting-state connectivity over the left parietal and temporal regions, but a U-shape development trajectory (decreased from neonate to 3 months and increased from 3 to 6 months) over frontal and occipital regions in infants aged between 0 to 6 months old (31). The connectivity over the frontal regions continued to develop from childhood to adulthood, with Eng et al. finding increasing prefrontal cortex connectivity from 4 to 5 years old (38); and Cai et al. finding increasing nodal properties (i.e., nodal degree, nodal efficiency, number of frontal hubs) from 7 to 27 years (30). On the other hand, the default mode network (DMN, synchronized activation between the medial prefrontal cortex and the temporoparietal junction) showed an inverted U-shaped developmental trajectory, with the connectivity peaking at 2 years old (32).

The developmental trajectories of preterm infants and children with or at elevated risk for developing ASD and dyslexia differed from those of their neurotypical peers (Supplementary Table S4). For example, Watanabe et al. found similar developmental changes in hPod (in-phase to anti-phase) in preterm and full-term infants during the first 2 months of age, but the development in early preterm infants preceded that of late preterm and full-term infants and progressed at a slower pace (39). In infants with an elevated risk for developing ASD (e.g., those with a family history of ASD), there tended to be an initial outgrowth followed by a later decrease in functional connectivity compared to their neurotypical peers (19, 20). Specifically, Keehn et al. found greater intrinsic (i.e., cortical connectivity when task-related signal fluctuations were removed) and co-activation connectivity (i.e., cortical connectivity when task-related activation was not removed) at 3 months but decreased connectivity at 12 months in high-risk compared to low-risk infants (19), while Zhang et al. found greater frontal and temporal connectivity in high-risk than low-risk infants at 5 months but not at 10 months (20). During childhood, children with ASD showed a U-shaped developmental trajectory of connectivity over the left middle frontal gyrus and age-related decreases in nodal metrics over the right temporoparietal junction, which is opposed to their TD peers (21). Lastly, while TD children showed age-related increases in activation over the prefrontal cortex, children with dyslexia failed to show similar developmental changes in the prefrontal cortex (25).

#### 3.4.2. Visual information processing, social and non-social

Nineteen studies investigated the neurodevelopmental trajectories of visual information processing, with 5 studies focused on non-social stimuli (34, 41–44), 5 compared stimuli with different levels of social information (14, 45–48), 7 targeted facial information (49–55), and 2 focused on the humour and empathy components in social contexts (56, 57) (Supplementary Table S5). For *non-social information processing*, Taga et al. found localized occipital lobe activation when receiving visual stimuli during awake and global activation over frontal, temporal, and occipital lobes during sleep (34). Moreover, there was a developmental trend of reduced occipital lobe activation from 3 to 6 months old when receiving visual stimuli during sleep (34). Watanabe et al. and Wilcox et al. studied the effects of object characteristics (e.g., color, shape, speed) on neural activity (41, 42). When viewing colourful vs. black and white stimuli, 2-month-old infants showed similar hemodynamic changes while 3-month-old infants showed greater anterior and middle occipital activation when viewing colourful vs. black and white stimuli (41). In addition, there is a developmental trend of decreased posterior parietal activation when viewing objects with different shapes, and a trend of decreased anterior temporal activation when viewing objects with different speeds between 3 and 12 months old (42). Lastly, Rodriguez et al. studied the visual orientation attention in infants (4 ~ 12 months old) (43) while Hirial et al. studied the visual perspective later in life (7 to 16 years old) (44). In the first year of life, infants moved from left-lateralized to bilateral prefrontal and parietal activation during a visual orientation attention task (43). During childhood, younger children (7 ~ 11 years old) showed higher superior temporal, angular gyrus, and frontal activation when taking other’s than their own perspective, while older children showed similar hemodynamic changes during both visual perspective tasks (44) (Supplementary Table S5).

For *different levels of social information*, 4 studies from 2 research groups compared infants’ hemodynamic changes when processing social (i.e., facial and hand movements) vs. non-social information (i.e., moving toys and pictures of transportation) (14, 45–47). They found greater activation over bilateral temporal–parietal junctions during social than non-social information processing, consistently in infants aged between 4 to 36 months (14, 45–47). Hukuno et al. further found greater right angular gyrus activation during congruent (i.e., timely, synchronized) than incongruent (i.e., 3 s delayed) social interactions in 6 ~ 8 and 10 ~ 12 months old infants, with no significant age-related changes found between the age groups (48). These findings suggest that the temporal–parietal junctions are sensitive to the levels of social stimuli, developed early on in life, and remain stable throughout the first few years of life (Supplementary Table S5).

For *facial information processing*, four studies investigated the neurodevelopmental trajectories when processing faces with different spatial arrangements and orientations (Canonical vs. Scrumble: Honda et al.; Upright vs. Inverted: Kobayashi et al.; Frontal vs. Profile: Nakato et al. and Ichikawa et al.) (49–52). These studies suggested the important role of temporal regions in face processing as they are sensitive to face spatial arrangements and orientations (49–52). Moreover, profile face processing develops between 5 and 6 months old, which is later than frontal face processing (develops before 3 months old) (51) and inverted face processing (develops before 5 months old) (50). Another two studies focused on the processing of familiar vs. unfamiliar faces (53, 54). Timeo et al. found similar hemodynamic changes when processing faces with their own and different ethnicities in 5-month-old infants, but greater occipital-temporal activation in different than their own ethnicity in the 9-months own infants, suggesting increased specialization over occipital-temporal regions (53). The trend of increasing specialization remains till puberty but decreased again in adulthood. Takamura et al. found greater frontal lobe activation when viewing smiles of their own vs. unfamiliar mothers in 9–14 years old boys, but the difference was not significant in adults (54). Lastly, one study focused on facial emotion processing found differentiated hemodynamic changes over the frontal and temporal regions when processing happy, fearful, and angry faces, with no age-related differences between 5 to 7 months old infants (55). In short, the temporal regions are important for face processing, and become more sensitive/specific to different facial characteristics (i.e., face orientation and familiarity) during development (Supplementary Table S5).

Beyond processing the characteristics of social stimuli, two studies investigate the perception of empathy and humor components in social contexts (56, 57). Brink et al. found greater Orbitofrontal and Dorsolateral Prefrontal Cortex (DLPFC) activation when viewing empathy-embedded vs. neutral cartoon pictures in children between 4 to 8 years old, with older children (6 ~ 8 years) showing greater DLPFC activation when watching empathy pictures compared to younger children (4 ~ 6 years) (57). On the other hand, Mayseless et al. recorded the hemodynamic changes when children aged between 6 to 8 years view funny and neural video clips (56). The children showed greater activation over left Temporo-Occipito-Parietal Junction (TOPJ), Inferior Parietal Lobe (IPL), DLPFC, and right Inferior Frontal Gyrus (IFG) during funny vs. neutral video watching, with the Left TOPJ activation positively correlated with age (56). These results suggest that the higher cognitive demanding components, such as the perception of empathy and humour, continue to develop during childhood (Supplementary Table S5).

#### 3.4.3. Auditory information processing and language

A total of 30 studies have explored the developmental trajectories for auditory information, language processing, and language productions (14, 28, 34, 45, 46, 57–80), with 2 solely focused on non-social auditory stimuli, 5 examined social vs. non-social stimuli, 18 focused exclusively on social and language stimuli, and 5 centered on language expression. For *non-social auditory processing*, infants exhibited better localized and differentiated hemodynamic changes during their first year of life (28, 58) (Supplementary Table S6). For instance, at 3 months old, infants showed widespread activation over bilateral prefrontal, temporal, and temporoparietal regions when exposed to long and short random tone sequences during sleep, while at 6 months old, they exhibited more localized activation over the temporal regions (58). Additionally, 3 months old infants displayed similar hemodynamic changes when listening to fast and slow acoustic modulations, while 6 months old infants demonstrated differentiated cortical lateralization when exposed to different stimuli (Fast: Left lateralized inferior temporal and right lateralized temporoparietal activation; Slow: Right lateralized inferior frontal and temporoparietal activation) (28). When comparing *social vs. non-social* auditory information processing, infants had greater hemodynamic changes when exposed to social than non-social stimuli, with the differences increasing with age (14, 59, 60) (Supplementary Table S6). Specifically, Lloyd-Fox et al. observed greater temporal lobe activation in 9 to 24-month-old infants when listening to *social vs. non-social* sounds, but not in infants aged 0 to 8 months (14); Grossmann et al. found greater bilateral temporal activation when listening to vocal vs. non-vocal sounds in 7-month-old infants but not in those aged 4 months (59); and Sato et al. found left -lateralized activation when processing words vs. pure tone in 10-month-old infants but not in those aged 4 months (60). However, the developmental trend of increased social-related hemodynamic changes were not consistent in infants and children living in adverse environments, suggesting that exposure to psychosocial adversity might affect the neurodevelopmental trajectory in processing auditory social information (45, 46).

During the typical development of *social auditory and language information processing*, infants and children exhibit increased sensitivity to stimuli that carry greater social and language values (61, 62). They also switched from recruiting bilateral auditory circuits to a linguistic circuit that is more left hemisphere dominant (61, 63–67). Although some studies failed to find age-related differences during their targeted age range (Zimmermann et al., 1 ~ 11 weeks; Taga et al., 3 ~ 6 months; Minagawa-Kawai et al., 3 ~ 14 months) (34, 68, 69), Minagawa-Kawai et al. found similar temporal activation in 3 to 11-month-old infants but greater temporal lobe activation when processing pseudowords that varied in linguistic characteristic than in vowel duration in infants aged 13 to 28 months old (61). Similarly, while 3-month-old infants showed similar hemodynamic changes over bilateral temporal region, 6 months old infants showed more focal changes in HbO and HbR responses to communicative (i.e., human communicative and infant-directed sounds) vs. non-communicative sounds (62). Moreover, several studies found age-related increases in left-lateralization over the language circuit (i.e., frontal, temporal regions) during infancy when processing pseudowords (61), forward/backward sentences (63), and native/non-native speech (64, 65), with the left-lateralization continuing into adulthood (67). Besides the increased left-lateralization of language network, infants and children also exhibited age-related differences in the sensitivity to novelty (70–74) and emotion imposed in the social auditory/language stimuli (57, 75). For example, Lloyd-Fox et al. found more robust habituation and recovery of response to novelty over the middle and superior temporal regions in 8-month-old than 5-month-old infants (71); Minagawa et al. found greater IFG and premotor activation when listening to familiar than nonfamiliar speech in 7 to 10-month-old but not 5 to 6-month-old infants (72); Yamasaki et al. found greater temporal activation when processing sentences read by mothers than by strangers in 3 to 4.5-year-old but not 4.5 to 6-year-old children (73). For vocal emotion processing, Zhao et al. found age-related increases in sensitivity over the right temporal lobe in infants aged 6 to 12 months when processing angry vocalization (75), while Brink et al. found greater left IFG activation in 6 to 8-year-old than 4 to 6-year-old children when listening to affective empathy stories (57).

Five studies focusing on *expressive language* development in school-age children have suggested similar developmental trajectories to those observed in receptive language studies (22, 76–79). For example, Goto et al. found an age-related increase in left-lateralization over DLPFC when repeating auditory stimuli in reverted order in children aged 7 to 12 years old (76). Additionally, 3 studies using the verbal fluency task found age-related increases in bilateral Broca’s and Wernicke’s areas (77), as well as in the frontal lobes in school-age children (22, 77, 78). Although the findings are consistent, the developmental trajectory of expressive language may be influenced by early language experience (79). Jasińska et al. used a single-word reading paradigm (including regular, irregular, and nonsense spelling) and found greater and more variable neural activation over bilateral IFG, Superior Temporal Gyrus (STG), and Prefrontal Cortex (PFC) regions in bilingual vs. unilingual children (79).

#### 3.4.4. Tactile information processing

There are three articles that investigated the neurodevelopmental trajectories of tactile processing during the first year of life (81–83) (Supplementary Table S7). De Oliveira et al. provided vibrotactile stimuli on preterm and full-term infants’ right hands and recorded their hemodynamic changes over frontal, parietal, and temporal regions at 6 and 12 months (81). The results suggest different developmental trajectories but generally more localized activation over the sensorimotor areas in preterm (i.e., bilateral to contralateral activation) and full-term infants (i.e., more localized contralateral activation) (81). Kida et al. and Miguel et al. investigated the hemodynamic changes in TD infants when processing different types of tactile information (82, 83). Greater activation over bilateral anterior PFC during pleasant touch compared to neural touch in 10-month-old infants, while no differentiated activation was found in 3- and 6-months old infants (82). When providing affective and discrimination touches, greater differentiate activation between the types of tactile inputs and increased recruitment of the temporal region for affective touch was found in 12-month compared to 7-month-old infants (83). Please see detailed study designs and main findings in Supplementary Table S7.

#### 3.4.5. Executive functioning

The developmental trajectories of executive functioning have been studied in three main categories, namely inhibitory control, mental flexibility, and working memory (Supplementary Table S8). For *inhibitory control*, 8 articles examined the prefrontal hemodynamic responses in children and adults during Flanker, Stroop, go/no-go, rock-paper-scissor, and multi-source interference tasks (23, 24, 84–89). TD children were found to have age-related increases in prefrontal activation in response to Flankers, Stroop, and go/no-go tasks, from childhood to adulthood (23, 84, 86, 87). Similarly, Papasideri et al. reported that age was a significant moderator of the relationship between bilateral medial PFC and general anxiety disorder, and the neural activity over this brain region predicted the anxiety, depression, and negative affect more strongly in older adolescents than in the younger ones (89). However, age-related differences were less consistent in children with obesity and ADHD (23, 24, 85). For example, Huang et al. failed to find an age-related increase in prefrontal cortex activation in obese adolescents (mean age 16.11y ± 4.75y) during a Stroop task (85). Although Yasumura et al. found a significant correlation between age and frontal hemodynamic activity during a Stroop task in children with ADHD (23), Ishii et al. found similar frontal lobe activation in children with ADHD aged between 8 to 14 years (24). Besides obesity and ADHD symptoms, education status may also affect developmental trajectories (88). McKay et al. found a longitudinal increase in the differences in parietal activation between the go/no-go conditions in children with a year of formal schooling, but a longitudinal decrease in the differences in same-age children who remained in kindergarten (88).

*Working memory* was examined in 5 studies (Supplementary Table S8) (90–94). Similar to the developmental trajectories in inhibitory control, there was an age-related increase in prefrontal activation in TD children (90, 91). Perlman et al. measured prefrontal hemodynamic responses in 3- to 7-year-old children during a spatial working memory task and reported that prefrontal activation during both short and long working memory blocks was positively correlated with age (90); Kawakubo et al. found a positive correlation between age and activation in the left prefrontal cortex in healthy non-affected siblings with ASD, but not in individuals with ASD nor healthy controls (91). Moreover, Buss et al. found that 4-year-olds had a more robust response in the parietal cortex during a change detection task than 3-year-olds (92). Besides the age-related increase in PFC activation, increased right-lateralization over the PFC was found in school-age children (93, 94). Specifically, Tsujii et al. found greater right lateralization over PFC in 7 to 8-year-old than in 5 to 6-year-olds, and the right lateralization in 5- to 6-year-olds could predict spatial working memory performance at 7 to 8 years (93). Suzuki et al. also observed more robust right lateralization over PFC in 11- to 12-year-olds than in 7- to 10-year-olds (94).

Lastly, *Mental flexibility* was investigated in children and adults in 3 articles conducted by the same research group using a dimensional change card sort task (Supplementary Table S8) (26, 27, 95). Through a longitudinal study on children aged 3 to 5 years old, Moriguchi et al. demonstrated an age-related increase in bilateral inferior prefrontal activation during both pre-switch and post-switch phases (26). The authors also found similar findings in a cross-sectional study, where they observed more robust lateral prefrontal activation during observation and execution phases in 4- to 6-year-olds compared to 3- to 4-year-olds, who showed significant lateral prefrontal activation during observation but not execution phases (27). The age-related increase in prefrontal cortex activation may reach a plateau at 5 years old, as Moriguchi et al. observed an adult-like activation in 5-year-old children (95).

#### 3.4.6. Motor tasks and others

Three cross-sectional studies have delved into the neurodevelopmental trajectories of motor skills (Supplementary Table S9) (96–98). Infants, when acquiring goal-directed motor experience, exhibited more localized and greater activity over the primary motor cortex (96). Specifically, Nishiyori et al. found that 12-month-old infants showed more localized and a greater change in HbO concentration during reaching and more widespread but a greater HbO responses during stepping than 6-month-old infants (96). From school age to adulthood, children demonstrated increased left-lateralization during motor tasks and different hemodynamic patterns when learning from live versus televised models (97, 98). Specifically, Su et al. measured hemodynamic responses in the frontal, temporal, and parietal cortex, and found greater right hemispheric activation during action observation, but less left hemispheric activation during action and imitation in children compared to adults (97). On the other hand, Moriguchi et al. found greater left primary cortex activation in children when learning from live vs. televised models, whereas adults showed similar bilateral primary cortex activation regardless of the types of the learning model (98).

Three other studies examined the development of arithmetic (99), gratification (100), and creativity (101) (Supplementary Table S9). Although the activation over the parietal region was sensitive to the math questions presented in words vs. numbers (99), and the right inferior prefrontal cortex was sensitive to immediate vs. delayed gratification (100), the neural activity did not differ between age groups. On the contrary, Saggar et al. (101) found that lateral frontal lobe segregation and specialization were associated with different creativity developmental trajectories in school-age children (101).

## 4. Discussion

### 4.1. Summary of main findings

The current systematic review provides a comprehensive summary of 84 original studies that have utilized fNIRS to investigate the developmental trajectories in typically developing infants and children, as well as individuals with (or at a greater risk for) developmental disorders (e.g., ASD, ADHD, Dyslexia). The findings indicate an age-related increase in resting-state connectivity and leftward network asymmetry in typically developing infants and children, with distinct developmental trajectories observed in different cortical regions. Moreover, during the processing of visual, auditory, and tactile information, we observed more localized and differentiated hemodynamic changes from infancy than childhood, suggesting better specialized and response-specific neural networks. In addition, as children develop language and executive functioning skills, we observed an increased left-lateralization of the temporal and frontal networks, as well as an increase in prefrontal activation. Notably, these developmental trajectories might be affected by a variety of factors, including birth histories, demographic characteristics, diagnoses, experience, and environmental factors. The absence of a standardized experimental setup and uniform data-processing/analyzing protocols further compounds the challenge of generalizing findings. Consequently, there is a distinct need for additional research endeavors aimed at proposing standardized methodologies for data collection and analysis, validating these developmental trajectories, delving into the factors that contribute to them, and exploring the potential utilization of these neurobiomarkers in both early identification of developmental disorders and in monitoring intervention outcomes.

### 4.2. Age-related differences in phase oscillation and increases in global/local efficiency, interhemispheric connectivity, leftward network asymmetry during resting-state

The resting-state studies in the current systematic review suggested age-related changes in phase difference between HbO and HbR oscillations (34) and increases in global/local efficiency (29, 30), interhemispheric connectivity (31, 32), leftward network asymmetry during resting state (29, 33). Specifically, the studies on phase difference between oscillation have shown a shift of hPod from in-phase to anti-phase, along with an age-related increase in hPod_L_ (34–37). hPod referred to the time-averaged phase differences between the instantaneous phases of HbO abd HbR and is said to be a good indicator of the development changes in the physiological mechanisms for circulatory, metabolic, and neurovascular functions (39). It is possible that the rapid change of hPod and hPod_L_ in the first year of life might reflect the rapid adaptation of the regulation of circular systems (e.g., lung and heart) to the extrauterine environment (39) as well as the development toward a mature form of neurovascular coupling (102). hPod_L_, on the other hand, referred to the strength of the coupling imposed in hPod (39). The age-related changes in hPod_L_ were different between cortical regions, suggest potential differential development within distinct areas of the brain (35). It’s important to acknowledge that these differences could be influenced by a range of factors (e.g., environmental) that contribute to the complex developmental landscape of the brain.

Local and global efficiency are considered good indicators of network segregation and integration and is associated with various cognitive functions (103). Our fNIRS findings on the age-related increase in global and/or local efficiency are consistent with some of the MRI and EEG studies and may reflect a neural maturation process of cognitive functions (104–106). Similarly, the age-related increase in interhemispheric connectivity observed in this systematic review is in agreement with previous fMRI findings, suggesting the functional maturation of the corpus callosum (31, 32, 107). Lastly, the age-related increase of leftward network asymmetry is well documented in fMRI studies (108). Reynolds et al. conducted an analysis of 386 fMRI data sets from 117 typically developing children and revealed increased leftward asymmetry over the inferior frontal gyrus in 2 ~ 7.5-year-olds (108). The developmental increase in leftward network asymmetry found in this fNIRS systematic review aligns with the fMRI findings and may reflect the maturation of the language network (29, 33).

Despite a general trend toward increased network segregation/integration, research using fNIRS has suggested that distinct developmental trajectories occur across cortical regions (30–32, 38). Similar region-specific variations in development were also found in resting-state fMRI studies (109–111). For example, Zhang et al. proposed a maturation order that starts with primary functional systems (e.g., primary visual cortex), processing to the mediation systems (e.g., temporal and parietal regions), and culminates in the higher cognitive systems (e.g., prefrontal cortex) during infancy (111). Later in childhood, Chen et al. observed an early developmental increase in connectivity over the default mode network and cingulo-opercular network, followed by a later increase in the fronto-parietal network (109). The fNIRS studies included in the systematic review also identified an early development of default mode network connectivity, with peak connectivity occurring approximately at 2 years of age (32); as well as a age-related increase in the connectivity over the frontal regions throughout childhood (30, 38), supporting the order of system maturation.

### 4.3. More localized and differentiate hemodynamic changes when processing visual, auditory, and tactile stimuli

In this systematic review, we present evidence of localized and increased differentiated hemodynamic changes during the processing of visual (41, 51, 53, 56, 57), auditory (58–60, 62, 75), and tactile information (81–83). This finding supports the notion that infants undergo interactive specialization of neural networks in the first few years of life (112). During this period brain regions become increasingly specialized and only respond to selective stimuli (112). This specialization process may occur due to selective pruning of synaptic connections and inhibition of alternative pathways (113, 114). A study that gathered 90 longitudinal fMRI data sets also suggested a developmental trend from widespread to more localized sensorimotor pathways in full-term and preterm infants during the first year of life (115).

The fNIRS studies in the current systematic review are consistent with the developmental trend of specialized neural networks, with increased differentiated hemodynamic changes associated with various stimuli characteristics, the familiarity of the stimuli, as well as the level of social and emotional information imposed on the stimuli in the first year of life. For instance, during visual information processing, Watanabe et al. found greater occipital activation in 3-month-old infants when viewing colorful vs. black and white stimuli, while 2-month-olds showed no differentiation across stimuli (41). Timeo et al. found greater occipital-temporal activation in 9-month-old infants when processing African (unfamiliar to the infants) vs. Caucasian (familiar) faces, while 5-month-olds showed no differentiate activation in response to face familiarity (53). During auditory information processing, Grossman et al. found greater superior temporal activation in 7-month-old infants when processing vocal vs. non-vocal sounds, while 4-month-olds showed no differentiate activation across stimuli (59). During tactile information processing, Kida et al. found greater bilateral prefrontal cortex activation in 10-month-old infants during pleasant vs. neutral touch, while 3- and 6-months olds showed no such differentiation (82). The sensory processing network continues to mature during childhood, as Brink et al. found that children aged 6 to 8 years old had greater dorsolateral prefrontal cortex activation when viewing and greater left inferior frontal lobe activation when listening to affective empathy stories compared to children aged between 4 to 6 years old (57). In short, the findings from the current fNIRS studies are consistent with the interactive specialization framework which showed localized and differentiated hemodynamic changes when processing multiple types of stimuli.

### 4.4. Developmental trend of increasing left-lateralization over temporal and frontal regions during language processing and expression

The current systematic review suggests an age-related increase in left lateralization over the temporal and frontal regions during language processing and expression, suggesting a transition from a bilateral auditory circuit to a left-lateralized language circuit during language development (Figure 4) (61, 63–65, 76). This finding aligns with the results of fMRI and EEG studies. A systematic review of 39 fMRI studies suggested increased frontal and temporal lobe lateralization associated with receptive and expressive language in childhood, with language lateralization established approximately around age 5 (3). Using fMRI, Olulade et al. further suggested bilateral activation during 4 to 6 years of age, followed by a decrease in activation over the right homolog of Broca’s area during childhood (6 to 13 years), leading to increased left-lateralization over the language circuits (116). In addition, an EEG study found significant left negativity of N150 and N350 during automatic word processing in adults, but not children around 10 years old, indicating immature word recognition and phonological processing at this age (117). While most of the fMRI and EEG studies have focused on toddlers and school-age children, numerous fNIRS studies have reported similar developmental trends in the left-lateralized language circuit in the first year of life (61, 63–65). For example, Minakawa-Kawai et al. found left lateralized temporal lobe activation during phonemic processing in infants over 12 months old, but not in infants aged between 3 to 11 months (61). Similarly, Petitto et al. found greater left lateralization over the frontal lobe in infants aged 10 to 12 months compared to those aged between 4- to 6-month-old when processing forward vs. backward sentences (64). In summary, the fNIRS studies included in the current systematic review are consistent with previous fMRI and EEG studies, and they further extend the neurodevelopmental findings to an earlier age.

### 4.5. Age-related increases in prefrontal cortex activation during executive functioning tasks

In the current systematic review, developmental increase in prefrontal hemodynamic activity was reported in three domains of executive functioning, including inhibition control (23, 84, 86, 87, 89), working memory (90, 91), and mental flexibility in typically developing children (Figure 4) (26, 27, 95). This finding from studies using fNIRS is consistent with studies using EEG and fMRI (118, 119). Measurement of event-related potential of executive functioning indicated age-related decrease of P300 latency- an indicator or neural speed and efficiency and increase of P300 amplitude- an indicator of growing cognitive resources (118). Similarly, studies using fMRI have shown developmental increases of BOLD signal during different inhibition control tasks (119). In addition to the age-related prefrontal activity increase, studies using fNIRS have indicated a developmental alteration of brain lateralization. Specifically, right lateralization was reported to be more robust in older children than younger ones during working memory task (93, 94). This age-related increase of the right lateralization was also found in an fMRI study during a memory-guided saccade task (120). In general, findings on developmental trajectory in executive functioning using fNIRS agreed with findings using fMRI and EEG.

### 4.6. Atypical developmental trajectories in children with developmental disorders

Compared to typically developing children, children with developmental disorders, such as ASD (19–22), ADHD (23, 24), and Dyslexia (25), demonstrate atypical neurodevelopmental trajectories during resting-state and when performing executive functioning tasks. Studies using fNIRS indicate that children with ASD show an initial outgrowth followed by a later decrease in resting-state functional connectivity (19, 20). For example, infants who had an elevated risk for developing ASD demonstrated greater functional connectivity at 3 months but lower connectivity at 12 months compared to their typically developing peers (19). Although the results are inconsistent, the initial overconnectivity and later underconnectivity in children with ASD have also been reported in EEG and fMRI studies, which may be explained by the short-range overconnectivity and long-range underconnectivity (121, 122). On the other hand, children with ASD and ADHD exhibit lower prefrontal activation compared to their typically developing peers throughout childhood (23, 24, 91). Although atypical prefrontal cortex activation associated with executive functioning was found in children with ASD and ADHD across life span using fMRI and EEG (103, 123, 124), more longitudinal studies are needed to document the developmental trajectories in children with developmental disorders.

### 4.7. Recommendations for future research and clinical implications

The present systematic review provides support for the use of fNIRS in investigating neurodevelopmental trajectories in both typically developing children and children with developmental disorders. Nonetheless, there are some limitations in the current fNIRS studies. For example, only a small proportion of studies (18.4%) employed a longitudinal study design, whereas the majority of studies employed a cross-sectional design that compared neural findings between age groups. Unlike longitudinal studies, cross-sectional studies are susceptible to individual variabilities and do not establish causal relationships for neurodevelopment. To minimize the variability between subjects, it is important for more studies to adopt a longitudinal approach to track the developmental trajectories of the same group of children. With respect to the targeted population, only 15.4% of the studies included children with developmental disorders (or those at higher risk of developing the disorders). Therefore, future research is required to examine different developmental trajectories between typically developing children and children with developmental disorders, as well as to investigate how environmental or demographic characteristics may impact children’s developmental trajectories. In terms of experimental setups, only 20% of the studies used spatial registration methods to validate their fNIRS system coverage. Furthermore, the experimental set-up (task parameters, probe placements), processing, and analyzing procedures varied across studies, limiting the ability to generalize and compare findings across studies. Future studies should work on the data collection and processing pipeline and suggest the standard way of analyzing/reporting fNIRS findings.

Given the rapid growth of head size during development, future studies should use spatial registration techniques and refer to MRI for better localization. Additionally, many fNIRS studies reported high exclusion rates, particularly those involving younger infants (Figure 3). To make fNIRS experiment more tolerable for participating infants, future researchers should work on reducing the weight of the fNIRS cap and shortening the experimental procedures. Lastly, since fNIRS is a relatively novel neuroimaging tool, it’s crucial to validate its findings against those of fMRI and EEG techniques. For example, future researchers could consider using multimodal techniques, such as simultaneous recording of EEG and fNIRS or the Hepta-scan environment (e.g., simultaneous recording of fNIRS, blood pressure, anesthesia monitoring, as well as magnetic resonance encephalography with scalp electroencephalography), to validate fNIRS findings (125–128). Please see Table 1 for the summary of recommendations for future research.

**Table 1:** Summary of clinical implications and recommendations for future research.

Recommendation for future research	Clinical implications
To minimize variability between subjects, more studies should utilize a longitudinal study design to monitor the developmental trajectories of the same group of subjects. Additional studies are required to investigate different developmental trajectories between typically developing children and children with developmental disorders. Environmental (e.g., adversity) and demographic factors (e.g., gender) should be examined to determine their influence on children’s developmental trajectories. Spatial registration methods should be used in future studies to achieve more accurate localization of targeted brain regions. Researchers should focus on developing more infant-friendly fNIRS caps (e.g., reducing weight) to decrease exclusion rates. To validate fNIRS findings, multimodal techniques such as simultaneous recording of fNIRS and EEG should be utilized.	Although further studies are required to validate the findings, potential neurobiomarkers reported in the current systematic review could aid in the early identification of developmental disorders. Clinicians could consider using these neurobiomarkers in predicting the children’s performance, monitoring the progress of symptoms, and investigating the intervention effects.

In terms of clinical implications, it’s essential to acknowledge that additional studies are required to validate the findings and propose a consistent and reliable methodology for extracting/calculating fNIRS variables. Nonetheless, this systematic review underscores prospective neurobiomarkers that could play a pivotal role in the premature detection of developmental disorders. An illustrative example can be found in the observed preliminary overconnectivity (i.e., higher correlation of averaged time course between channels/region of interest) among children with ASD, which could potentially serve as a neurobiomarker aiding in the early identification of this disorder (129). Clinicians could also consider using these neurobiomarkers to predict the children’s performance, tracking the progress of symptoms, and investigate the effects of interventions. In fact, a recent systematic review has shown significant neural effects of movement interventions in children with developmental disorders, supporting the use of fNIRS, along with other neuroimaging tools, for monitoring the intervention outcomes (127). Please refer to Table 1 for the summary of clinical implications.

## 5. Conclusion

To the best of our knowledge, this is the first systematic review to summarize fNIRS-related developmental trajectories in typically developing children and children with developmental disorders. Our findings suggest a general developmental trend toward increased network integration/segregation, interhemispheric connectivity, and leftward network asymmetry during resting-state; more localized and specific neural responses to visual, auditory, and tactile stimuli; increased left-lateralized language network; as well as greater prefrontal activation during executive functioning tasks. The developmental trajectories are different in children with developmental disorders and may be influenced by birth histories, demographic characteristics, experience, and other environmental factors. Although the fNIRS findings align with those of EEG and fMRI, it is imperative to exercise additional caution in generalizing the fNIRS results, owing to the relatively restricted number of studies and the absence of established standardized data collection and analysis protocols. Furthermore, there exists a pressing need for supplementary studies that can validate the neurodevelopmental trajectories and delve into the prospective utility of these neurobiomarkers in the timely detection of developmental disorders.

## Author contributions

W-CS was in charge of the initial design, searching, screening, data extraction, and writing the first draft of the manuscript. RC and NA participated in the screening and writing progress. TN wrote part of the manuscript and provided revisions to the manuscript. TG contributed to the quality assessment and the final revision of the manuscript. AG was in charge of overall direction and planning, supervised the project, and provided final approval. All authors contributed to the article and approved the submitted version.

## Funding

This study was supported by the Intramural Research Program (IRP) of the National Institute of Child Health and Human Development (Project Number: 1ZIAHD008882-10), the National Institute of Health’s Bench-to-Bedside Program.

## Conflict of interest

The authors declare that the research was conducted in the absence of any commercial or financial relationships that could be construed as a potential conflict of interest.

## Publisher’s note

All claims expressed in this article are solely those of the authors and do not necessarily represent those of their affiliated organizations, or those of the publisher, the editors and the reviewers. Any product that may be evaluated in this article, or claim that may be made by its manufacturer, is not guaranteed or endorsed by the publisher.

## Footnotes

^1^ https://homer-fnirs.org/
