# Supplementary material for: The use of functional near-infrared spectroscopy in tracking neurodevelopmental trajectories in infants and children with or without developmental disorders: a systematic review
Source: Front Psychiatry. 2023 Sep 14;14:1210000. doi: 10.3389/fpsyt.2023.1210000 (PMC10536152; doi:10.3389/fpsyt.2023.1210000)
Supplement: Supplementary file 1 [file Data_Sheet_1.docx]

Supplementary Material

The use of Functional Near-Infrared Spectroscopy in Tracking Neurodevelopmental Trajectories in Children with or without Developmental Disorders: A Systematic Review

**Wan-Chun Su,^1^ Rebekah Colacot,^1^ Nora Ahmed,^1^ Thien Nguyen,^1^ Tony George^1^, Amir Gandjbakhche ^1*^**

*** Correspondence:** Amir Gandjbakhche, gandjbaa@mail.nih.gov

# Supplementary Figure

**Supplementary Figure S1**. Common data processing pipeline for fNIRS variables.


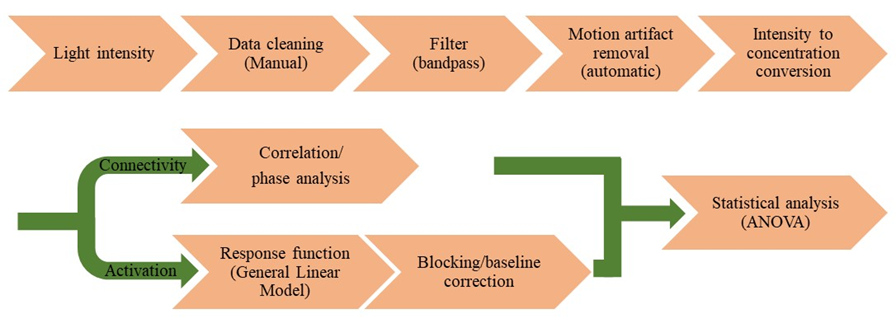


# Supplementary Table

**Supplementary Table S1.** Search terms for different databases

| **PubMed** | **PsycINFO** | **Scopus** | **Web of Science** |
| --- | --- | --- | --- |
| ("spectroscopy, near infrared"[MeSH Terms] OR "NIRS"[All Fields] OR "NIR"[All Fields] OR "near-infrared spectroscopy"[All Fields]) AND ("Longitudinal Studies"[MeSH Terms] OR "Longitudinal"[All Fields] OR "Longitudinally"[All Fields] OR "Age groups"[All Fields] OR “trajectory”[All fields] OR “Trajectories”[All fields] OR "Developmental"[Title/Abstract] OR "Development"[Title]) | (NIRS OR NIR OR near-infrared spectroscopy) AND (Longitudinal OR Longitudinally OR Age groups OR trajectory OR Trajectories OR tiab(Developmental) OR title(Development)) | TITLE-ABS(“nirs” OR “NIR” OR “near infrared spectroscopy”) AND TITLE-ABS(“Longitudinal” OR “Longitudinally” OR “Age groups” OR “Trajectory” OR “Trajectories” OR “developmental”) | (TI=(NIRS) OR TI=(NIR)) AND (TI=(Longitudinal) OR TI=(Longitudinally) OR TI=(Age groups) OR TI=(Trajectory) OR TI=(Trajectories) OR TI=(Developmental) OR TI=(Development)) |

**Supplementary Table S2.** Criteria for the Quality Assessment Tool for Observational Cohort and Cross-Sectional Study from the National Institute of Health.

| **Criteria** | **Yes** | **No** | **Other (CD, NR, NA)** |
| --- | --- | --- | --- |
| 1. Was the research question or objective in this paper clearly stated? |  |  |  |
| 2. Was the study population clearly specified and defined? |  |  |  |
| 3. Was the participation rate of eligible persons at least 50%? |  |  |  |
| 4. Were all the subjects selected or recruited from the same or similar populations (including the same time period)? Were inclusion and exclusion criteria for being in the study prespecified and applied uniformly to all participants? |  |  |  |
| 5. Was a sample size justification, power description, or variance and effect estimates provided? |  |  |  |
| 6. For the analyses in this paper, were the exposure(s) of interest measured prior to the outcome(s) being measured? |  |  |  |
| 7. Was the timeframe sufficient so that one could reasonably expect to see an association between exposure and outcome if it existed? |  |  |  |
| 8. For exposures that can vary in amount or level, did the study examine different levels of the exposure as related to the outcome (e.g., categories of exposure, or exposure measured as continuous variable)? |  |  |  |
| 9. Were the exposure measures (independent variables) clearly defined, valid, reliable, and implemented consistently across all study participants? |  |  |  |
| 10. Was the exposure(s) assessed more than once over time? |  |  |  |
| 11. Were the outcome measures (dependent variables) clearly defined, valid, reliable, and implemented consistently across all study participants? |  |  |  |
| 12. Were the outcome assessors blinded to the exposure status of participants? |  |  |  |
| 13. Was loss to follow-up after baseline 20% or less? |  |  |  |
| 14. Were key potential confounding variables measured and adjusted statistically for their impact on the relationship between exposure(s) and outcome(s)? |  |  |  |

Link to the assessment criteria: <https://www.nhlbi.nih.gov/health-topics/study-quality-assessment-tools>)

**Supplementary Table S3.** Quality assessment for the included articles

|  | Author, Year | 1 | 2 | 3 | 4 | 5 | 6 | 7 | 8 | 9 | 10 | 11 | 12 | 13 | 14 | % | Rating |
| --- | --- | --- | --- | --- | --- | --- | --- | --- | --- | --- | --- | --- | --- | --- | --- | --- | --- |
| *1* | *Homase, 2010* | Y | N | N | N | N | Y | Y | Y | Y | N | Y | - | - | N | 50 | Poor |
| *2* | *Taga, 2018* | Y | N | Y | N | N | Y | Y | Y | Y | N | Y | - | - | N | 58 | Poor |
| *3* | *Taga 2018* | Y | Y | Y | N | N | Y | Y | Y | Y | N | Y | - | - | N | 67 | Fair |
| *4* | *Liu, 2022* | Y | Y | Y | N | N | Y | Y | Y | Y | N | Y | - | - | Y | 75 | Good |
| *5* | *Bulgarelli, 2020* | Y | N | N | Y | N | Y | Y | Y | Y | N | Y | - | - | N | 58 | Poor |
| *6* | *Eng, 2022* | Y | Y | Y | N | N | Y | Y | Y | Y | Y | Y | - | N | Y | 77 | Good |
| *7* | *Cai, 2018* | Y | N | Y | N | N | Y | Y | Y | Y | N | Y | - | - | N | 58 | Poor |
| *8* | *Cai, 2019* | Y | N | Y | N | N | Y | Y | Y | Y | N | Y | - | - | Y | 67 | Fair |
| *9* | *Watanabe, 2017* | Y | Y | Y | N | N | Y | Y | Y | Y | N | Y | - | - | N | 67 | Fair |
| *10* | *Fanceschini, 2007* | Y | Y | Y | N | N | Y | Y | Y | Y | N | Y | - | - | N | 67 | Fair |
| *11* | *Liang, 2021* | Y | Y | Y | N | N | Y | Y | Y | Y | 5 | Y | - | - | N | 73 | Good |
| *12* | *Liang, 2022* | Y | Y | Y | N | N | Y | Y | Y | Y | N | Y | - | - | N | 67 | Fair |
| *13* | *Keehn, 2013* | Y | N | N | N | N | Y | Y | Y | Y | N | Y | - | - | Y | 58 | Poor |
| *14* | *Zhang, 2022* | Y | N | Y | N | N | Y | Y | Y | Y | N | Y | - | - | Y | 67 | Fair |
| *15* | *Cao, 2021* | Y | Y | Y | N | N | Y | Y | Y | Y | N | Y | - | - | N | 67 | Fair |
| *16* | *Pecyna, 2013* | Y | N | Y | N | N | Y | Y | Y | Y | N | Y | - | - | N | 58 | Poor |
| *17* | *Watanabe, 2010* | Y | Y | N | N | N | Y | Y | Y | Y | N | Y | - | - | N | 58 | Poor |
| *18* | *Wilcox, 2014* | Y | Y | Y | N | N | Y | Y | Y | Y | N | Y | - | - | N | 67 | Fair |
| *19* | *Rodriguez, 2020* | Y | N | Y | N | N | Y | Y | Y | Y | N | Y | - | - | N | 58 | Poor |
| *20* | *Hirai, 2022* | Y | Y | Y | N | N | Y | Y | Y | Y | N | Y | - | - | N | 67 | Fair |
| *21* | *Lloyd-Fox, 2017* | Y | Y | N | Y | N | Y | Y | Y | Y | N | Y | - | - | N | 67 | Fair |
| *22* | *Pirazzoli, 2022* | Y | Y | Y | N | N | Y | Y | Y | Y | N | Y | - | - | N | 67 | Fair |
| *23* | *Perdue, 2019* | Y | Y | Y | N | N | Y | Y | Y | Y | N | Y | - | - | N | 67 | Fair |
| *24* | *Van Der Kant, 2018* | Y | N | Y | N | N | Y | Y | N | Y | N | Y | - | - | N | 50 | Poor |
| *25* | *Hakuno, 2020* | Y | Y | N | N | N | Y | Y | Y | Y | N | Y | - | - | N | 58 | Poor |
| *26* | *Honda, 2010* | Y | N | Y | N | N | Y | Y | Y | Y | N | Y | - | - | N | 58 | Poor |
| *27* | *Kobayashi, 2021* | Y | Y | Y | N | Y | Y | Y | Y | Y | N | Y | - | - | N | 75 | Good |
| *28* | *Ichikawa, 2019* | Y | N | Y | N | N | Y | Y | Y | Y | N | Y | - | - | N | 58 | Poor |
| *29* | *Nakato, 2009* | Y | N | Y | N | N | Y | Y | Y | Y | N | Y | - | - | N | 58 | Poor |
| *30* | *Timeo, 2019* | Y | N | Y | N | N | Y | Y | Y | Y | N | Y | - | - | N | 58 | Poor |
| *31* | *Takamura, 2015* | Y | Y | Y | N | N | Y | Y | Y | Y | N | Y | - | - | N | 67 | Fair |
| *32* | *Bayet, 2021* | Y | N | Y | N | N | Y | Y | Y | Y | N | Y | - | - | N | 58 | Poor |
| *33* | *Brink, 2011* | Y | Y | Y | N | N | Y | Y | Y | Y | N | Y | - | - | N | 67 | Fair |
| *34* | *Mayseless, 2021* | Y | N | Y | N | Y | Y | Y | Y | Y | N | Y | - | - | Y | 75 | Good |
| *35* | *Homae, 2012* | Y | N | Y | N | N | Y | Y | Y | Y | N | Y | - | - | N | 58 | Poor |
| *36* | *Telkemeyer, 2011* | Y | N | Y | N | N | Y | Y | Y | Y | N | Y | - | - | N | 58 | Poor |
| *37* | *Grossmann, 2010* | Y | N | Y | N | N | Y | Y | Y | Y | N | Y | - | - | N | 58 | Poor |
| *38* | *Sato, 2010* | Y | N | Y | N | N | Y | Y | Y | Y | N | Y | - | - | N | 58 | Poor |
| *39* | *Zimmermann, 2012* | Y | Y | Y | N | N | Y | Y | Y | Y | N | Y | - | Y | N | 69 | Fair |
| *40* | *Minagawa, 2007* | Y | N | N | N | N | Y | Y | Y | Y | N | Y | - | - | N | 50 | Poor |
| *41* | *McDonald, 2019* | Y | Y | Y | Y | N | Y | Y | Y | Y | N | Y | - | N | N | 69 | Fair |
| *42* | *Zhang, 2022* | Y | N | Y | N | N | Y | Y | Y | Y | N | Y | - | - | N | 58 | Poor |
| *43* | *Petitto, 2012* | Y | N | Y | N | N | Y | Y | Y | Y | N | Y | - | - | N | 58 | Fair |
| *44* | *Fava, 2014* | Y | Y | Y | N | N | Y | Y | Y | Y | N | Y | - | - | N | 67 | Fair |
| *45* | *Minagawa-Kawai, 2013* | Y | N | Y | N | N | Y | Y | Y | Y | N | Y | - | - | N | 58 | Poor |
| *46* | *Yamane, 2021* | Y | Y | N | N | N | Y | Y | Y | Y | N | Y | - | - | N | 58 | Poor |
| *47* | *Sela, 2011* | Y | N | Y | N | N | Y | Y | N | Y | N | Y | - | - | N | 50 | Poor |
| *48* | *Ding, 2021* | Y | N | Y | N | N | Y | Y | N | Y | N | Y | - | - | Y | 58 | Poor |
| *49* | *Wagner, 2011* | Y | N | Y | N | N | Y | Y | Y | Y | N | Y | - | - | N | 58 | Poor |
| *50* | *Loyd-Fox, 2019* | Y | Y | - | Y | N | Y | Y | Y | Y | N | Y | - | - | Y | 82 | Strong |
| *51* | *Minagawa, 2017* | Y | N | N | N | N | Y | Y | Y | Y | N | Y | - | - | N | 50 | Poor |
| *52* | *Yamasaki, 2013* | Y | N | Y | N | N | Y | Y | Y | Y | N | Y | - | - | N | 58 | Poor |
| *53* | *van der Kant, 2020* | Y | Y | Y | N | N | Y | Y | Y | Y | N | Y | - | - | N | 67 | Fair |
| *54* | *Zhao, 2021* | Y | Y | Y | N | N | Y | Y | Y | Y | N | Y | - | Y | N | 69 | Fair |
| *55* | *Goto, 2015* | Y | Y | Y | N | N | Y | Y | Y | Y | N | Y | - | - | N | 67 | Fair |
| *56* | *Paquette, 2015* | Y | N | Y | Y | N | Y | Y | N | Y | N | Y | - | - | N | 58 | Poor |
| *57* | *Kawakubo, 2011* | Y | Y | Y | Y | N | Y | Y | N | Y | N | Y | - | - | N | 67 | Fair |
| *58* | *Tando, 2014* | Y | N | Y | N | N | Y | Y | N | Y | N | Y | - | - | N | 50 | Poor |
| *59* | *Jasinska, 2014* | Y | N | Y | Y | N | Y | Y | N | Y | N | Y | - | - | Y | 67 | Fair |
| *60* | *Olivera. 2019* | Y | Y | Y | Y | N | Y | Y | Y | Y | N | Y | - | N | N | 69 | Fair |
| *61* | *Kida, 2013* | Y | N | Y | N | N | Y | Y | Y | Y | N | Y | - | - | N | 58 | Poor |
| *62* | *Miguel, 2019* | Y | Y | Y | N | N | Y | Y | Y | Y | N | Y | - | N | N | 62 | Fair |
| *63* | *Yeung et al., 2020* | Y | Y | Y | N | N | Y | Y | Y | Y | N | Y | - | - | N | 67 | Fair |
| *64* | *Schroeter, 2004* | Y | N | Y | Y | N | Y | Y | Y | Y | N | Y | - | - | N | 67 | Fair |
| *65* | *Mehnert, 2013* | Y | N | Y | N | N | Y | Y | Y | Y | N | Y | - | - | N | 58 | Poor |
| *66* | *Papasideris, 2021* | Y | Y | Y | N | N | Y | Y | Y | Y | N | Y | - | - | N | 67 | Fair |
| *67* | *Huang, 2019* | Y | Y | Y | N | N | Y | Y | Y | Y | N | Y | - | - | Y | 75 | Good |
| *68* | *Yasumura, 2019* | Y | Y | Y | Y | N | Y | Y | Y | Y | N | Y | - | - | N | 75 | Good |
| *69* | *Ishii, 2017* | Y | N | Y | N | N | Y | Y | N | Y | N | Y | - | - | Y | 58 | Poor |
| *70* | *McKay, 2022* | Y | Y | Y | N | N | Y | Y | N | Y | N | Y | - | Y | N | 62 | Fair |
| *71* | *Perlman, 2016* | Y | N | Y | N | N | Y | Y | Y | Y | N | Y | - | - | N | 58 | Poor |
| *72* | *Kawakubo, 2009* | Y | Y | Y | Y | N | Y | Y | Y | Y | N | Y | - | - | Y | 83 | Strong |
| *73* | *Buss, 2014* | Y | Y | Y | N | N | Y | Y | Y | Y | N | Y | - | - | N | 67 | Fair |
| *74* | *Tsujii, 2009* | Y | N | Y | N | N | Y | Y | Y | Y | N | Y | - | - | N | 58 | Poor |
| *75* | *Suzuki, 2018* | Y | N | Y | N | N | Y | Y | Y | Y | N | Y | - | - | N | 58 | Poor |
| *76* | *Moriguchi, 2011* | Y | N | Y | N | N | Y | Y | N | Y | N | Y | - | Y | N | 54 | Poor |
| *77* | *Moriguchi, 2020* | Y | Y | Y | N | N | Y | Y | Y | Y | N | Y | - | - | N | 67 | Fair |
| *78* | *Moriguchi, 2009* | N | N | Y | N | N | Y | Y | Y | Y | N | Y | - | - | N | 50 | Poor |
| *79* | *Nishiyori, 2016* | Y | N | Y | N | N | Y | Y | Y | Y | N | Y | - | - | N | 58 | Poor |
| *80* | *Moriguchi, 2014* | Y | N | Y | N | N | Y | Y | N | Y | N | Y | - | - | N | 50 | Poor |
| *81* | *Su, 2020* | Y | Y | Y | Y | N | Y | Y | N | Y | N | Y | N | - | N | 62 | Fair |
| *82* | *Obersteiner, 2010* | Y | N | Y | N | N | Y | Y | Y | Y | N | Y | - | - | N | 58 | Poor |
| *83* | *Moriguchi, 2018* | Y | Y | Y | N | N | Y | Y | Y | Y | N | Y | - | N | N | 62 | Fair |
| *84* | *Saggar, 2019* | Y | Y | Y | N | N | Y | Y | Y | Y | N | Y | - | Y | N | 69 | Fair |

**Supplementary Table S4. Study designs and main findings for the neurodevelopmental trajectory during resting-state**

| References | Design | # of visit | # of subject | Age  (range/ M ± SD) | Diagnoses/ Conditions | Tasks | Measures | Main findings |
| --- | --- | --- | --- | --- | --- | --- | --- | --- |
| Homae, 2010 (31) | C | 1 | 15  21  16 | 2~11d  3m  6m | TD | Resting state (sleep) | F, T, P, O | - Increased interhemispheric connectivity over temporal, parietal and occipital lobe from neonate to 6m.  - Increased L parietal and temporal connectivity from neonate to 6 m.  - U-shape development in frontal and occipital connectivity (decrease from neonate to 3m, and increase from 3~6m) |
| Taga, 2018a  (35) | C | 1 | 15  21  16 | 2~11d  3m  6m | TD | Resting state (sleep) | F, T, P, O | -hPod values over F, T, P, O lobes shifted from in-phase to antiphase between neonates and 3 m, and lasted until 6 m.  -hPod_L_ over P and O increased between neonates and 3~6 m, indicating increased phase locking |
| Taga, 2018b  (34) | C | 1 | 25  28  24  14 | 2.4~3.3m  3.4~4.3m  4.4~5.7m  9.7~11.0m | TD | Resting state (sleep), Visual and auditory stimuli (sleep & awake) | F, T, O | - Stronger connectivity, closer to anti-phase hPoD, larger hPoD_L_ during sleep vs. awake.  - Weaker temporal connectivity in older vs. younger groups.  - hPoD values over frontal, L temporal and occipital regions were closer to anti-phase and lager hPoD_L_ value over frontal and L temporal regions in older vs younger groups. |
| Liu, 2022  (29) | C | 1 | 35  31 | 3-6m  6-9m | TD | Resting state (sleep) | F, T, P, O | - Increased leftward asymmetry over the frontal and temporal regions from 3~6 to 6~9m  - Similar local efficiency but greater global efficiency over the left hemisphere in 6~9 vs 3~6m |
| Bulgarelli, 2020 (32) | L | 5 | 11  21  25  28  32 | 11.4m ± 0.3  18.5m ± 0.3  24.6m ± 0.5  30.6m ± 0.3  36.7m ± 0.5 | TD | Resting state (video) | F, T, P | - Connectivity over DMN (mPFC- TPJ) peaked at 24m  - Interhemispheric and intra hemispheric connectivity over L hemisphere (IFG, MTG, STG) showed significant changes between 11~18m, and remain stable afterward. |
| Eng, 2022 (38) | L | 4 | 41  41  40  30 | 4-5 y  72hr FU  1 m FU  4 m FU | TD | Resting state (viewed nonsocial & nonverbal stimuli | F | - High test-retest reliability of functional connectivity within 72h  - Functional connectivity over PFC increased between 4-5 y  - Functional connectivity predict later executive functioning performance. |
| Cai, 2018 (30) | C | 1 | 30  30  30 | 7~9y  11~13y  19~27y | TD | Resting state (eyes closed) | F, T, P, O | - Increased global efficacy from childhood to adolescent  - Increased clustering coefficient and local efficiency from adolescent to adulthood.  - Age-related increase in nodal properties (i.e., nodal degree, nodal efficiency, # of frontal hubs) over the frontal cortex from childhood to adulthood. |
| Cai, 2019 (33) | C | 1 | 30  30 | 7~9y  19~27y | TD | Resting state (eyes closed) | F, T, P, O | - Age-related increased leftward asymmetry in local efficiency from childhood to adulthood.  - Age-related increased leftward asymmetry in nodal efficiency over the frontal, parietal-occipital junction, and occipital region from childhood to adulthood. |
| Watanabe, 2017 (39) | C | 1 | 28  21  41  33  9  6  23  22  17  54 | 0-7w  8-13w  14-21w  0-7w  8-13w  14-21w  0-7w  8-13w  14-21w  8-16w | FT (GA:37-41w)  LPT (GA: 34-36w)  EPT (GA < 34w)  TD | Resting state (sleep) | F, T, O | - FT, LPT, EPT showed similar decrease in hPod (in-phase to anti-phase) in the first 2m  - Developmental changes of hPod in EPT precede LPT and FT but progressed at slower pace. |
| Franceschini_2007 (40) | C | 1 | 47 | 0-50 w | FT, PT | Resting state | F, T, P, O | -From 0-6 w HbT and StO_2_ significantly decrease while CBV remains constant in all regions (R and L temporal, R and L parietal, occipital and frontal)  -From 12-50 w HbT, StO_2_, and CBV significantly increase for most regions |
| Liang, 2021 (36) | C | 1 | 21  20  27  21  20  27 | -6.0~5.1w  -2.6~1.5w  3~4m  6~11y  19~27y  58~77y | PT  FT  TD | Resting state (Sleep) | F, T | - Increased phase difference from newborns to 3~4m and decreased in elderly age.  - Greatest phase locking index in childhood and adulthood and decreased in elderly age. |
| Liang, 2022 (37) | C | 1 | 38  23  35  21  18  27 | -6.0~5.1w  -2.6~1.5w  3~4m  6~11y  19~27y  58~77y | PT  FT  TD | Resting state  (Sleep in PT, Eye open in 3~4m, Eye close in children, adult, and elderly) | F, T | - STDE: greater STDE in 3~4 m compared to neonate groups. The STDE value peaked at 6~11y and decreased during elderly age.  - NGSC & SampEn: Similar values during infancy. The values increased from childhood to adulthood and decreased during elderly age. |
| Keehn, 2013 (19) | L | 4 | 17  12  8  6  13  18  21  21 | 3m,  6m,  9m,  12m  3m,  6m,  9m,  12m | HR-ASD(ASD siblings)  LR-ASD | Facial processing, language processing (trisyllabic sequence) | T | -3m: greater intrinsic and co-activation connectivity in HR-ASD vs LR-ASD  -6m and 9m: no significant group differences.  - 12m: Lower intrinsic and co-activation connectivity in HR-ASD vs LR-ASD |
| Zhang, 2022 (20) | C | 1 | 26  19  23  15 | 5m  10m  5m  10m | HR-ASD  (ASD siblings)    LR-ASD | Resting state (Awake) | F, T, P | -5 & 10m: Greater frontal, temporal functional connectivity in HR-ASD vs LR-ASD  -5 m but not 10m: Greater local and nodal efficiency in HR-ASD vs LR-ASD, suggesting overgrowth local network. |
| Cao et al., 2021 (21) | C | 1 | 77    40 | 6~16y | ASD (Medical records, ADOS)  TD | Resting state (eyes closed) | F, T, P | - Similar global efficacy and age-related decrease in mPFC connectivity between TD and ASD groups.  - ASD: U-shaped trajectory over L MFG and age-related decrease of nodal metrics over R TPJ.  - TD: Inverted U-shaped trajectory over L MFG and age-related increase of nodal metrics over R TPJ. |
| Pecyna, 2013 (25) | C | 1 | 16  16  16  16  16  16 | 6-7y  9-10y  19-20y  6-7y  9-10y  19-20y | Dyslexic  TD | Resting state | F | - 6-7y TD showed a trend of greater left PFC activation compared to 9~10y and 19~20y.  -In Dyslexic group, there is no significant age-related difference in PFC.  -All dyslexic age groups had lower PFC activation compared to TD. |

C = Cross-sectional; L = Longitudinal; h = hour; d = day; w = week; m = month; y = year; FU = Follow up; M = mean; SD = standard deviation; L = left; R = right; F = frontal; T = temporal; P = parietal; O = occipital; TPJ = temporal-parietal junctions; PFC = prefrontal cortex; m PFC = medial prefrontal cortex; TD = typically developing; ASD = autism spectrum disorder; HR-ASD = high risk ASD; LR-ASD = low risk ASD; PT = preterm; EPT = early preterm; LPT = late preterm; FT = full-term; HPod = Hemoglobin phase; HPoD_L_ = Phase locking index of hPod; PFC = Prefrontal Cortex; R = right; MFG = middle frontal gyrus; STG = superior temporal gyrus; MTG = middle temporal gyrus; DMN = Default Mode Network; HR-ASD: High-risk ASD; LR-ASD: Low-risk ASD; SampEn = Sample entropy; NGSC = Normalized global spatial complexity; STDE = Spatial time delay entropy; HbT = total hemoglobin concentrations; StO_2_ = oxygen saturation; CBV = cerebral blood volume. ADOS = Autism Diagnostic Observation Schedule.

**Supplementary Table S5. Study designs and main findings for the neurodevelopmental trajectory during visual information processing**

| References | Design | # of visit | # of subject | Age | Diagnoses/  Conditions | Tasks | Measures | Main findings |
| --- | --- | --- | --- | --- | --- | --- | --- | --- |
| Non-Social Information | | | | | | | | |
| Taga, 2018 (34) | C | 1 | 91 | 3m, 4m, 5m, 6m | TD | Visual stimuli when awake and during sleep | F, T, O | -Awake: Visual stimuli induce activation over occipital lobe  -Sleep: Visual stimuli induce global activation over frontal, temporal, and occipital regions  - Developmental trends of occipital lobe activation to deactivation from 3 to 6m during sleep. |
| Watanabe, 2010 (41) | C | 1 | 65 | 2m, 3m | TD | Colorful and black and white stimuli | F, O | -2m: Similar anterior and middle occipital lobe activation during both conditions  -3m: greater anterior and middle occipital lobe activation when viewing colorful vs black and white stimuli |
| Wilcox, 2014 (42) | C | 1 | 153 | 3m~6m, 7m~8m, 10m~12m | TD | Object shape and speed | Left T, P, O | -Shape: consistent anterior temporal activation from 3~12m, but only 3~6 m showed posterior parietal activation  -Speed: consistent posterior parietal activation from 3~12m, but only 3~8m showed anterior temporal activation. |
| Rodriguez, 2020 (43) | C | 1 | 27 | 4m, 8m, 12m | TD | Visual orientation attention | F, P | - Infants in all groups showed activation over right PFC and parietal regions during Visual orientation tasks  - Infants at 12 m additional showed left PFC and left parietal activation. |
| Hirai, 2022 (44) | C | 1 | 26 | 7~11y, 11~16y | TD | Visual perspective | F, T, P | - 7~11y: Higher superior temporal, angular gyrus, and frontal activation when taking other’s vs own perspective  - 11~16y: No significant difference between other’s vs own perspective. |
| Social Information | | | | | | | | |
| Lloyd-Fox, 2017 (14) | L, C | 1~3 | 16~25 | 4~8m, 9~13m, 12~16m, 18~24m | TD | Social vs nonsocial visual stimuli | F, T | - Consistently greater activation over posterior superior temporal and temporal parietal junction when watching social vs nonsocial stimuli across age groups. |
| Pirazzoli, 2022 (45) | L, C | 1~2 | 112~155 | 6m, 24m, 36m | TD, low source | Social vs nonsocial visual stimuli | F, T | - Consistently greater activation over B frontal and temporal-parietal junction when watching social vs nonsocial stimuli across age groups. |
| Perdue, 2019 (46) | C | 1 | 85, 105 | 6m, 36m | TD, low source | Social vs nonsocial visual stimuli | F, T | - Consistently greater activation over B frontal and temporal-parietal junction when watching social vs nonsocial stimuli across age groups.  - No significant differences between age groups |
| Van Der Kant, 2018 (47) | C | 1 | 37 | 5-8m | TD | Social (Hand and Face) vs non-social visual stimuli | F, T | - Greater right temporal activation during social vs non-social dynamic  - No age-related differences in cortical activation |
| Hakuno, 2020 (48) | C | 1 | 38 | 6~8m, 10~12m | TD | Congruent vs Incongruent social interaction | T | - 6~8m: Greater right AG activation during congruent vs incongruent  - 10~12m: Greater right STS-AG activation during congruent vs incongruent  - no significant different between age groups |
| Honda, 2010 (49) | C | 1 | 21 | 7m~8m, Adult | TD | Canonical vs Scrambled face | T, O | - Both infants and adults showed greater right temporal-occipital activation when viewing Canonical vs Scrambled faces  - The differences between Canonical vs Scrambled were greater in infants than adults. |
| Kobayashi, 2021 (50) | C | 1 | 32 | 5m~6m, 7m~8m | TD | Dynamic face vs body; Upright vs Inversion | T | -Dynamic Face: Broader activation temporal regions in 7~8m vs 5~6m  -Dynamic Body: no significant age difference  - Both 5~6m and 7~8 m infants showed greater activation during upright vs inverted body processing. |
| Ichikawa, 2019 (51) | L | 5 | 14 | 3m, 4m, 5m, 6m, 7m, 8m | TD | Frontal vs profile face | T | - 3m: Greater B temporal activation during frontal than profile face processing  - 3~8 m: Lager increases in R temporal activation during profile than frontal face processing  - Processing of profile faces emerged between 5-6 m |
| Nakato, 2009 (52) | C | 1 | 20 | 5m, 8m | TD | Frontal vs profile face | T | - 5 m: Increased R temporal lobe activation during frontal face processing only.  - 8 m: Increased R temporal lobe activation during both profile and frontal face processing. |
| Timeo, 2019 (53) | C | 1 | 30 | 5m, 9m | TD | Caucasian and African faces | T, O | - 5m: no significant difference between Caucasian and African faces  - 9m: greater occipital-temporal activation when processing African vs Caucasian faces |
| Takamura, 2015 (54) | C | 1 | 85 | 9 y, 14y, 20 y | TD | Face of mother vs not mother | F | - 9y: right PFC, own > unfamiliar mother  - 14y: left PFC, own > unfamiliar mother  - 20y: own ≈ unfamiliar mother |
| Bayet, 2021 (55) | C | 1 | 100 | 5m, 7m | TD | Happy, fearful, angry faces | F, T | - Frontal and temporal regions are sensitive to emotional expression  - No significant age-related changes in cortical activation. |
| Brink, 2011 (57) | C | 1 | 48 | 4~6.6 y, 6.6~8.8y | TD | Affective and Cognitive empathy stories | F, T, O | - Affective empathy vs neural: Increased Orbitofrontal and DLPFC activation, with 6~8 y showing greater DLPFC activation than the 4~6y.  - Cognitive empathy vs neutral: Increased Orbitofrontal activation, no age-related differences |
| Mayseless, 2021 (56) | C | 1 | 35 | 6~8y | TD | Funny vs Neutral video clips | F, T, P | - Greater activation over left TOPJ, IPL, DLPFC and right IFG during funny vs neutral.  - Left TOPJ activation positively correlated with age. |

C = Cross-sectional; L = Longitudinal; m = month; y = year; M = mean; SD = standard deviation; L = left; R = right; B = bilateral; TD = Typically Developing; V = visit; F = frontal; T = temporal; P = parietal; O = occipital; PFC = prefrontal cortex; DLPFC = dorsolateral prefrontal cortex; AG = angular gyrus; IFG = inferior frontal gyrus; IPL = inferior parietal lobe; STS = superior temporal sulcus; TOPJ = temporal-occipital-parietal junctions.

**Supplementary Table S6. Study designs and main findings for the neurodevelopmental trajectory during auditory information and language processing**

| References | Design | # of visit | # of subject | Age | Diagnoses/Conditions | Tasks | Measures | Main findings |
| --- | --- | --- | --- | --- | --- | --- | --- | --- |
| Non-Social auditory stimuli | | | | | | | | |
| Homae, 2012 (58) | C | 1 | 46 | 3m, 6m | TD | Long, short, random tone sequence during sleep | T | - 3m: Widespread activation over B PFC, temporal, temporoparietal regions. Greater R temporoparietal activation during random than long/short sequences.  - 6m: Localized activation over temporal regions. Greater R temporoparietal activation during short than random/long sequences. |
| Telkemeyer, 2011 (28) | C | 1 | 74 | 3m, 6m | TD | Fast and slow acoustic modulations | F, T, O | -3m: L lateralized superior frontal and posterior temporal activation during Fast and Slow conditions.  -6m: L lateralized inferior temporal and R lateralized temporoparietal activation during Fast condition; R lateralized inferior frontal and temporoparietal activation during Slow condition. |
| Social vs non-social auditory stimuli | | | | | | | | |
| Lloyd-Fox, 2017 (14) | L, C | 1~3 | 16~25 | 0~2m, 4~8m, 9~13m, 12~16m, 18~24m | TD | Social and non-social auditory stimuli | F, T | - 0~8 m: greater posterior temporal activation during non-social vs social  - 9~24m: greater anterior temporal activation during social vs non-social |
| Pirazzoli, 2022 (45) | L, C | 1~2 | 112~155 | 6m, 24m, 36m | TD, low source | Social vs nonsocial auditory stimuli | F, T | - 6m: greater L IFG and B posterior temporal activation during social vs non-social  - 24m: greater B IFG during social vs non-social  - 36m: no differences between social vs non-social |
| Perdue, 2019 (46) | C | 1 | ~85, 105 | 6m, 36m | TD, low source | Social vs nonsocial auditory stimuli | F, T | - 6m: greater B frontal & temporal activation during social vs non-social  - 36m: greater B middle temporal activation during social vs non-social  - No significant differences between age groups |
| Grossmann, 2010 (59) | C | 1 | 32 | 4m, 7m | TD | Vocal vs non-vocal sounds | F, T | - 4m: no significant differences between vocal vs non-vocal processing  - 7m: Greater B superior temporal cortex activation during vocal vs non-vocal processing. |
| Sato, 2010 (60) | C | 1 | 48 | 4m, 10m | TD | Words and pure tone | T | - 4 m: no significant differences when processing words and pure tone, and no significant lateralization  -10m: Left lateralization during word but not pure tone condition. |
| Social auditory stimuli and receptive language | | | | | | | | |
| Zimmermann, 2012 (68) | L | ~3 | 6 | 1-11w | Preterm infants | Repetitive Syllabus (e.g., ma, ma, ma) | Left T | - Inverted responses (negative HbO and positive HbR) were found between 3 to 8 weeks. |
| Taga, 2018 (34) | C | 4 | 91 | 3m, 4m, 5m, 6m | TD | Word processing during sleep and wakefulness | F, T, O | -Awake: Speech stimuli induce activation over B temporal lobes  -Sleep: Speech stimuli induce global activation over frontal, temporal, and occipital regions  - No significant age-related differences |
| Minagawa-Kawai, 2007 (61) | C | 1 | 57 | 3-4m  6-7m  10-11m  13-14m  25-28m | TD | Across -category (differ in linguistic) or within category (differ in vowel duration) phonemic processing | T | -3~4m and 10~11m: similar temporal activation during within and across category phonemic processing.  - 6~7m, 13~14m, and 25~28m: greater temporal lobe activation during across vs within category phonemic processing  - no cortical lateralization from 3~11m; Left lateralization over temporal lobe after 12m |
| McDonald, 2019 (62) | L | 2 | 42 | 3m, 6m | TD | Communicative, non-communicative, infant directed speech | F, T | -3 m: similar bilateral temporal activation when processing communicative and non-communicative human vocalizations.  -6 m: more focal responses to social sounds that carried increased social value |
| Zhang 2022 (63) | C | 1 | 40 | 5m,  10m | TD | Forward and backward sentence | F, T, P | -5m: no significant differences when processing forward vs backward sentences  -10m: left lateralized activation during forward vs backward sentence processing |
| Petitto, 2012 (64) | C | 1 | 61 | 4-6m, 10-12m | Bilingual & Monolingual TD | Native vs non-native phonetic processing | F, T | - Consistent left STG activation across ages and groups.  - Greater left lateralized IFG activation in older than younger children.  - Older but not younger bilingual children showed greater left IFG activation than Unilingual children. |
| Fava 2014 (65) | C | 1 | 35 | 3-6m  7~10m  11-14m | TD | Native vs Non-native speech | T | - 3~6m: Greater R temporal activation during non-native vs native  - 7~10m: no significant conditional differences.  - 11~14m: Greater L temporal activation during native vs non-native |
| Minagawa-Kawai, 2013 (69) | C | 1 | 86 | 3~5m, 6~7m, 10~11m, 12~14m | TD | Native vs Non-native phonological grammar processing | T | -No significant neural responses |
| Yamane, 2021 (66) | C | 1 | 25 | 4.5m, 12m | TD | Lyrics and Melodies | T | - 4.5m: B temporal activation during lyric-alone and similar R temporal activation during melody-alone and Lyrics and melody conditions.  - 12m: L temporal activation during lyric-alone, and greater B temporal activation during lyrics and melody compared to melody-alone condition. |
| Sela, 2011 (67) | C | 1 | 47 | 12y,  Adults | TD | Lexical Decision Task (Words vs Pseudowords) | F | - Both 12 y and adults showed lower minimum value and longer time to reach maximum value over L frontal lobe during pseudoword vs word.  - Children showed larger decline in the minimum value and reaches maximum value faster over L frontal lobe compared to the adults. |
| Ding 2021 (80) | C | 1 | 50 | 9~12y,  Adult | Unilingual and Bilingual TD | Syntactic processing of sentences | F | - Greater B DLPFC, MPFC, and L IPL in adults than 9~12y children. |
| Wagner, 2011 (70) | C | 1 | 28 | 7m, 9m | TD | Repetitive (e.g., ABB), and non-repetitive (e.g., ABC) grammar | T | -7m: Greater temporal lobe activation during ABB than ABC  -9m: Greater temporal lobe activation during ABC than ABB. |
| Lloyd-Fox, 2019 (71) | L | 2 | 120, 103 | 5m,8m | TD | Habituation of spoken sentence | F, T, P | - More robust habituation and recovery of response to novelty over middle and superior temporal regions from 5~8m |
| Minagawa, 2017 (72) | C | 1 | 54 | 5-6m,  7-8m,  9~10m | TD | Phonological short-term memory | T | - Training period: 7~8m and 9~10m (but not 5~6m) showed greater SMG activation during familiar than non-familiar condition  - Testing period: 7~8m and 9~10m (but not 5~6m) showed greater IFG and premotor activation during familiar than non-familiar condition |
| Yamasaki, 2013 (73) | C | 1 | 20 | 3~4.5y, 4.5~6y | TD | Familiar vs unfamiliar Linguistic processing | T | - 3~4.5y: greater temporal activation when processing mothers voice compared to unfamiliar voice and environment sounds.  4.5~6y: No significant differences between conditions.  - Younger children sowed greater temporal activation compared to older children when processing mother’s voice. |
| Van der Kant 2020 (74) | C | 1 | 50 | 2y,  3y | TD | Linguistic and non-linguistic non-adjacent dependency (NAD) learning | F, T, P | - 2 y: increased L temporal, inferior frontal, and parietal activation associated to the detection of linguistic NAD violation.  - 3y: increased B temporal and parietal activation associated to the detection of non-linguistic NAD violation. |
| Zhao, 2021 (75) | L | 3 | 40 | 6m, 9m, 12m | TD | Vocal Emotion processing | T | - Increased vocal anger sensitivity from 6-12 month over right temporal lobe  -Increased right temporal lobe activation from 6 to 12 month |
| Brink, 2011 (57) | C | 1 | 48 | 4~6.6 y, 6.6~8.8y | TD | Affective and Cognitive empathy stories | F, T, O | - Affective empathy vs Neutral: Greater left IFG activation in 6~8y than 4~6y  - Cognitive empathy vs Neutral: no age-related differences  - Affective vs cognitive empathy: Greater medial orbitofrontal activation in 6~8 than 4~6y |
| Expressive Language | | | | | | | | |
| Goto 2015 (76) | C | 1 | 103 | 7~8y,  9-10y,  11-12y | TD | Repeat or revert the stimuli | F | -7~8y: B DLPFC activation when repeating stimuli in a reverted order.  - 9~10y & 11~12y: left lateralized DLPFC activation when repeating stimuli in a reverted order. |
| Paquette, 2015 (77) | C | 1 | 40 | 3~6y, 7~10y, 11~16y,  19~30y | TD | Verbal fluency task | F, T, P | -Left lateralized activation over Broca’s and Wernicke’s area in all age groups.  - Age-related increases in B activation over Broca’s and Wernicke’s areas |
| Kawakubo 2011 (22) | C | 1 | 70 | 5~18y,  21-37y | TD | Verbal Fluency Test | F, T | - 5~18y: positive correlation between age and frontal lobe activation  - 21-37y: no significant correlation between age and frontal lobe activation. |
| Tando 2014 (78) | C | 1 | 46 | 6~8y, 9~11y, 12~14y, 15~18y, Adults | TD | Verbal Fluency Task | F | - Age-related increase in frontal lobe activation. |
| Jasińska 2014 (79) | C | 1 | 48 | 6~8y, 8~10y, Adults | Monolingual and bilingual TD | Regular, Irregular, and non-sense word reading | F | -6~8y: Similar L STG activation during Regular and Irregular word reading; Greater B IPL activation during regular vs nonsense word reading.  -8~10y: Greater L IFG activation during irregular vs regular but grater IPL activation during regular vs irregular word reading. Greater L IFG, B STG, B IPL activation during nonsense vs regular word reading.  * B IPL: Adults > children; L MTG: Children > Adults  * Bilingual TD had greater and more variable neural activation over B IFG, STG, and PFC. |

C = Cross-sectional; L = Longitudinal; w = week; m = month; y = year; M = mean; SD = standard deviation; L = left; R = right; B = bilateral; TD = Typically Developing; F = frontal; T = temporal; P = parietal; O = occipital; PFC = prefrontal cortex; DLPFC = dorsolateral prefrontal cortex; MPFC = medial prefrontal cortex; IFG = inferior frontal gyrus, STG = superior temporal gyrus; ; MTG = middle temporal gyrus; IPL = inferior parietal lobe; SMG = supramarginal gyrus.

**Supplementary Table S7. Study designs and main findings for the neurodevelopmental trajectory during tactile information processing**

| References | Design | # of visit | # of subject | Age | Diagnoses/ Conditions | Tasks | Measures | Main findings |
| --- | --- | --- | --- | --- | --- | --- | --- | --- |
| Tactile | | | | | | | | |
| De Oliveira, 2019 (81) | L | 2 | 22 | 6m, 12m | PT, FT | Vibrotactile stimulus | F, T, P, O | -Preterm: 6m: B activation over sensorimotor cortex; 12m: contralateral activation over sensorimotor cortex  -Full term: more localized contralateral activation over sensorimotor cortex from 6-12m |
| Kida, 2013 (82) | C | 1 | 32 | 3m, 6m, 10m | TD | Neutral vs pleasant touch | F | -3m & 6m: similar activation over B anterior PFC activation during neutral and pleasant touch  -10m: greater B anterior PFC activation during pleasant vs neutral touch |
| Miguel, 2019 (83) | L | 2 | 35  25 | 7m,  12m | TD | Affective vs discriminative touch | Left C, Right T | - Increased recruitment of the temporal region for affective touch from 7 to 12 m |

C = Cross-sectional; L = Longitudinal; m = month; B = bilateral; F = frontal; T = temporal; P = parietal; O = occipital; PFC = prefrontal cortex. PT = Preterm; FT = Full term.

**Supplementary Table S8.** Study designs and main findings for the neurodevelopmental trajectory when performing executive functioning tasks.

| References | Design | # of visit | # of subject | Age (M ± SD) | Diagnoses | Tasks | Measures | Main findings |
| --- | --- | --- | --- | --- | --- | --- | --- | --- |
| Inhibitory control | | | | | | | | |
| Yeung, 2020 (84) | C | 1 | 28 | 7~16y | TD | Flanker  Stroop | F | - During both flanker and Stroop tasks, the medial PFC activation significantly increased with age. |
| Schroeter, 2004 (86) | C | 1 | 23  14 | 7~13y  19~29y | TD | Stroop | F | - Hemodynamic responses in young adult occurred and peaked earlier than children  - Brain activation during Stroop in the dorsolateral PFC increased with age. |
| Mehnert, 2013 (87) | C | 1 | 22  20 | 4~6y,  21~36y | TD | Go/no-go | F, T, P | - Greater differences in frontal and parietal activation during no-go than go condition in adults vs. 4~6y  - In 4~6y children, age correlated significantly with the activation over right frontal lobe  - Within frontal cortex coherence was stronger and between frontal-parietal coherence was weaker in 4~6y compared to adults. |
| Papasideris, 2021 (89) | C | 1 | 67 | 13~18y | TD (28.4% depressive, 22.4% anxiety) | Multi-source interference (MSIT) | F | - Age was a significant moderator of the relationships between bilateral medial PFC activation and general anxiety disorder, depression, and negative affect  - Bilateral medial PFC predicted anxiety, depression, and negative affect more strongly for older than for younger adolescents. |
| Huang, 2019 (85) | C | 1 | 38 | 9~25y | TD (obese) | Stroop | F | - No age-related increased in PFC activation. |
| Yasumura, 2019 (23) | C | 1 | 68  72  38  29 | 6.5~9.9y  10.0~12.5y  6.8~9.9y  10.2~12.3y | TD  ADHD (DSM-IV, V) | Reverse Stroop | F | - 6~9y: TD children had significantly higher left PFC and middle PFC activation than ADHD children  - 10~12y: TD group had higher right PFC and middle PFC activation than ADHD group  - ADHD group: 10~12y children had higher left PFC function than 6~9y children  - TD group: 10~12 y children had higher middle and right PFC function than 6~9 y children |
| Ishii, 2017 (24) | C | 1 | 13  14  8  10 | 6~10y  11~16y  6~10y  11~16y | TD  ADHD (DSM-IV) | Rock-paper-scissor | F | - 6~10y: TD showed greater left lateral and medial frontal cortex activation than children with ADHD.  - 11~16y: TD showed grater left and right lateral and medial frontal activation than children with ADHD. |
| McKay, 2022 (88) | L | 2 | 80  (40 P1,  40 KG) | 4.5y±0.1  5.5y±0.1 | TD | Go/no-go | F, P | - P1: greater negative activation for response at incorrect no-go trials than for correct go trials in the bilateral middle frontal gyrus and bilateral IFG at both 4y and 5y.  - KG: difference in activation between go and no-go trials at 4y, but not 5y |
| Working Memory | | | | | | | | |
| Perlman, 2016 (90) | C | 1 | 68 | 3~7y | TD | Spatial WM (monkey banana tree) | F | - In both short and long WM, older children showed higher PFC activation than younger children.  - During the combined short and long WM blocks, age was positively correlated with the left lateral PFC activation. |
| Kawakubo, 2009 (91) | C | 1 | 14  13  12  12  14  13 | 5.8~16.6y  21.4~37.4y  5.9~15.8y  18.9~35.4y  6.6~17.3y  18.3~39.0y | TD  ASD-S  ASD (DSM-VI, CARS-2) | Letter fluency task | F | - Children: No significant difference among 3 groups  - Adult: ASDs had was significantly smaller PFC activation than controls  - Left PFC positively correlated with age only in ASD-S group. |
| Buss, 2014 (92) | C | 1 | 28  19 | 3y,  4y | TD | Change detection | F, P | - 4y had more robust response in the parietal cortex and better sensitivity to memory load manipulation compared to 3y. |
| Tsujii, 2009 (93) | L | 2 | 8 | 5~6y  7~8y | TD | Spatial item-recognition | F | - Greater right lateralization over PFC in 7~8y than 5~6y children.  - Children who have greater right lateralization over PFC at 5~6y responded more rapidly during the spatial working memory task at 7~8y |
| Suzuki, 2018 (94) | C | 1 | 25  36  17 | 7-8y  9-10y  11-12y | TD | Visuospatial WM | F | - 7~10y: right lateralized PFC activation during sequential but not spontaneous presentation.  11~12y: right lateralized PFC during both sequential and spontaneous presentation. |
| Mental Flexibility | | | | | | | | |
| Moriguchi, 2011 (26) | L | 2 | 15  13 | 3.1~3.9y  3.8~4.6y | TD | Dimensional change card sort | F | - Greater B inferior PFC during both pre-switch and post-switch phases at 4y than 3y  - Pass group: R inferior PFC at 3y and B inferior PFC at 4y  - Perseverate group: no significant inferior PFC activation at 3y but significant left inferior PFC activation at 4y |
| Moriguchi, 2020 (27) | C | 1 | 25  20 | 3.5y~4.5y,  4.7~6.5y | TD | Dimensional change card sort | F | - 3.5~4.5y: Significant PFC activation during execution but not observation phase.  -4.7~6.5y: Consistent PFC activation during both execution and observation phases. |
| Moriguchi, 2009 (95) | C | 1 | 15  11  10 | 3y  5y  21~31y | TD | Dimensional change card sort | F | -Adults, 5y, and 3y who passed the task: Significant activation in the bilateral inferior PFC was found during both pre-switch and post-switch phases.  - 3y with perseverative errors: no significant B inferior PFC during both pre-switch and post-switch phases. |

C = Cross-sectional; L = Longitudinal; y = year; R = right; B = bilateral; M = mean; SD = standard deviation; F = frontal; T = temporal; P = parietal; O = occipital; TD = typically developing; KG = Kindergarten; ASD = autism spectrum disorder; ASD-s = siblings of children with ASD; ADHD = Attention-Deficit/Hyperactivity Disorder; IFG = inferior frontal gyrus; PFC = prefrontal cortex; WM = working memory. CARS-2 = Childhood Autism Rating Scale-2^nd^ edition; DSM-IV = Diagnostic and Statistical Manual of Mental Disorders, Fourth Edition; DSM-V = Diagnostic and Statistical Manual of Mental Disorders, Fifth Edition.

.

**Supplementary Table S9.** Study designs and main findings for the neurodevelopmental trajectory when performing motor and other tasks

| References | Design | # of visit | # of subject | Age (M ± SD) | Diagnoses | Tasks | Measures | Main findings |
| --- | --- | --- | --- | --- | --- | --- | --- | --- |
| Motor | | | | | | | | |
| Nishiyori, 2016 (96) | C | 1 | 22 | 6m, 12m | TD | Reaching  Stepping | C | - Reaching: 12m showed more localized and greater activity over primary motor cortex compared to 6m.  - Stepping: 12m showed more widespread but greater activity over primary motor cortex compared to 6m. |
| Su, 2020 (97) | C | 1 | 17  15 | 10.8y ± 0.7  22.6y ± 0.7 | TD | Reach to cleanup | F, T, P | - Observation: children showed greater right hemispheric activation than adults  - Execution & Imitation: children exhibited less left hemispheric activation than adults |
| Moriguchi, 2014 (98) | C | 1 | 14  15 | 6.1y ± 0.6  25.3y ± 4.5 | TD | Learn to sort card from live vs televised model | C | - Children: greater left primary cortex activation during motor learning from live vs video demonstration.  - Adults: Similar bilateral primary cortex activation between live and video conditions. |
| Arithmetic | | | | | | | | |
| Obersteiner, 2010 (99) | C | 1 | 46  44 | 9.98y±0.49  13.88y±0.75 | TD | Addition (words vs number) | P | - Parietal brain activation was similar in grade 4 and 8 students as well as in students of different competency levels  - There was a slight increase of parietal activation when solving math problems presenting in words compared to numbers. |
| Gratification | | | | | | | | |
| Moriguchi, 2018 (100) | L | 2 | 99  45 | 5y±10.6m  5y6m±6.8m | TD | Gratification (immediate vs delayed) | F | - Greater activation of the right inferior PFC during the task phase than the rest phase in both age groups  - No age-related differences in PFC activation |
| Creativity | | | | | | | | |
| Saggar, 2019 (101) | L | 3 | 56 | 9.20 y ± 7.5 m | TD | Control and Creative drawing | F | - R lateral frontal lobe segregation and specialization is associated to different creativity developmental trajectories. |

C = Cross-sectional; L = Longitudinal; m = month; y = year; M = mean; SD = standard deviation; L = left; R = right; F = frontal; T = temporal; P = parietal; O = occipital; C = Central lobe, including primary motor area. PFC = prefrontal cortex.
